# Senescent fibroblasts modulate the radiation response of neighboring epithelial cells

**DOI:** 10.1038/s41420-025-02796-z

**Published:** 2025-10-20

**Authors:** Maja Buchholzki, Lisa Marie Stasch, Bettina Budeus, Vaanilaa Ketheeswaranathan, Zehra Fatma Sevindik, Verena Jendrossek, Diana Klein

**Affiliations:** Institute for Cell Biology (Cancer Research), Medical Faculty, University of Duisburg-Essen, Essen, Germany

**Keywords:** Senescence, Predictive markers, Respiratory distress syndrome, Cell death, Lung cancer

## Abstract

Radiation-induced lung injury (RILI) not only limits the therapeutic dose that can be administered during chest and thorax radiotherapy (RT), but also significantly impairs patients’ health and quality of life. RT-induced senescence and the associated altered secretory profile, the senescence-associated secretory phenotype (SASP), have emerged as a central process for the development and progression of pneumonitis and pulmonary fibrosis. Among various lung cell types, the phenomenon of permanent cell cycle arrest, which is also accompanied by significant morphological changes, has been observed especially in epithelial cells. RILI arises from a complex interplay of cell types and signaling pathways, but it has not yet been clarified when which lung cell types become senescent during RT and how induced changes in one cell type may influence senescence or the RT-dependent cell fate overall in another, adjacent cell type. Here, the different cellular fates particularly senescence versus apoptotic cell death following RT-induced genotoxic stress were investigated particularly in epithelial cells and fibroblasts providing further insights into the radiosensitivity of these lung cells. Fibroblasts that have become senescent during RT alter the RT response and thus the cell fate of co-cultured epithelial cells. In addition, new candidate genes were identified that were induced in the various cellular subpopulations of complex epithelial-fibroblast spheroids as an approximate in vivo cell culture model after irradiation. These candidate genes could be used in future studies as additional RT-induced gene sets and, in particular, as senescence-associated gene sets. A comprehensive understanding of the dynamic changes of these cellular components is crucial to specify new strategies for the prevention of RILI.

## Introduction

In the 1960s it was first discovered that human fibroblasts could only undergo a limited number of divisions before entering a state of irreversible growth arrest, primarily caused by telomere shortening and not by external stress, a phenomenon now known as replicative senescence [1, 2]. Telomere shortening is the reason that in a healthy organism, senescent cells gradually accumulate over time, particularly in aging tissues [3]. However, senescence can also be triggered by various intrinsic and extrinsic stimuli, including oncogene activation, loss of tumor suppressor genes, metabolic or genotoxic stressors, induced e.g., following exposure to radiation, a phenomenon known as premature senescence [3, 4]. This stable cell cycle arrest is characterized by an upregulation of cell cycle inhibitors, including p16INK4a, p21CIP1, and p27KIP1, alongside increased expression of p19ARF, p53, and PAI-1, which contribute to maintain the senescent state by reinforcing cell cycle arrest [5]. Morphologically, senescent cells exhibit a distinctive increase in size and a rounded, flattened shape, which further differentiates them from proliferating cells [3]. One central hallmark of senescent cells is the hyper-secretory phenotype, known as senescence-associated secretory phenotype (SASP), wherein the cells release high levels of pro-inflammatory cytokines, growth factors, and other bioactive factors into the surrounding microenvironment [6, 7]. The SASP is mainly regulated by key transcription factors such as NF-κB (nuclear factor ‘kappa-light-chain-enhancer’ of activated B-cells) [8] and C/EBPβ (CCAAT/enhancer-binding protein β) [9], and can act as a double-edged sword, with beneficial and damaging effects [10–12]. Concerning the latter one, SASP components such as monocyte chemoattractant proteins (MCPs), macrophage inflammatory proteins (MIPs), and interleukins (e.g., IL-6 and IL-8) can promote chronic inflammation, angiogenesis and tumor progression, and can induce senescence in neighboring cells, thereby amplifying the senescence response within a tissue highlighting its detrimental potential [11–13].

Radiation-induced lung injury (RILI) is a serious treatment complication, that affects patients who receive thoracic radiotherapy (RT) or are preconditioned with total body irradiation for bone marrow transplantation. Approximately 10–30% of these patients may develop radiation-induced pneumonitis as a subacute treatment-related toxicity and/or radiation-induced pulmonary fibrosis as a late toxicity [14, 15]. There is only a symptomatic, not yet a causal treatment for RILI, which is ultimately due to the fact that unfortunately the mechanisms by which radiation causes RILI are not yet fully understood [16, 17]. There is increasing evidence that radiation-induced senescence may play an important and even central role in RILI, and that limiting the extent of senescence and/or eliminating senescent cells with senomorphic or senolytic agents represents a promising new therapeutic strategy for RILI [18–20]. Neutralizing certain SASP factors may also be effective in reducing radiation-induced normal tissue defects in the lung [20, 21]. However, since the induction of senescence in the lung depends on the pathological trigger (e.g., dose and fractionation in RT) as well as the duration of observation, the exact role of the possible senescent cell types remains unclear. In addition to the influence of individual senescent epithelial cell types [22–24], the senescence of fibroblasts, possibly also endothelial cells and infiltrating immune cells, is also being discussed [13, 25, 26]. Here we investigated radiation-induced senescence of fibroblasts and epithelial cells in order to specify potential interactions that could presumably improve the understanding of the clinical picture of RILI, but could also serve as potential prognostic and/or therapeutic targets.

## Results

**Fig. 1:**
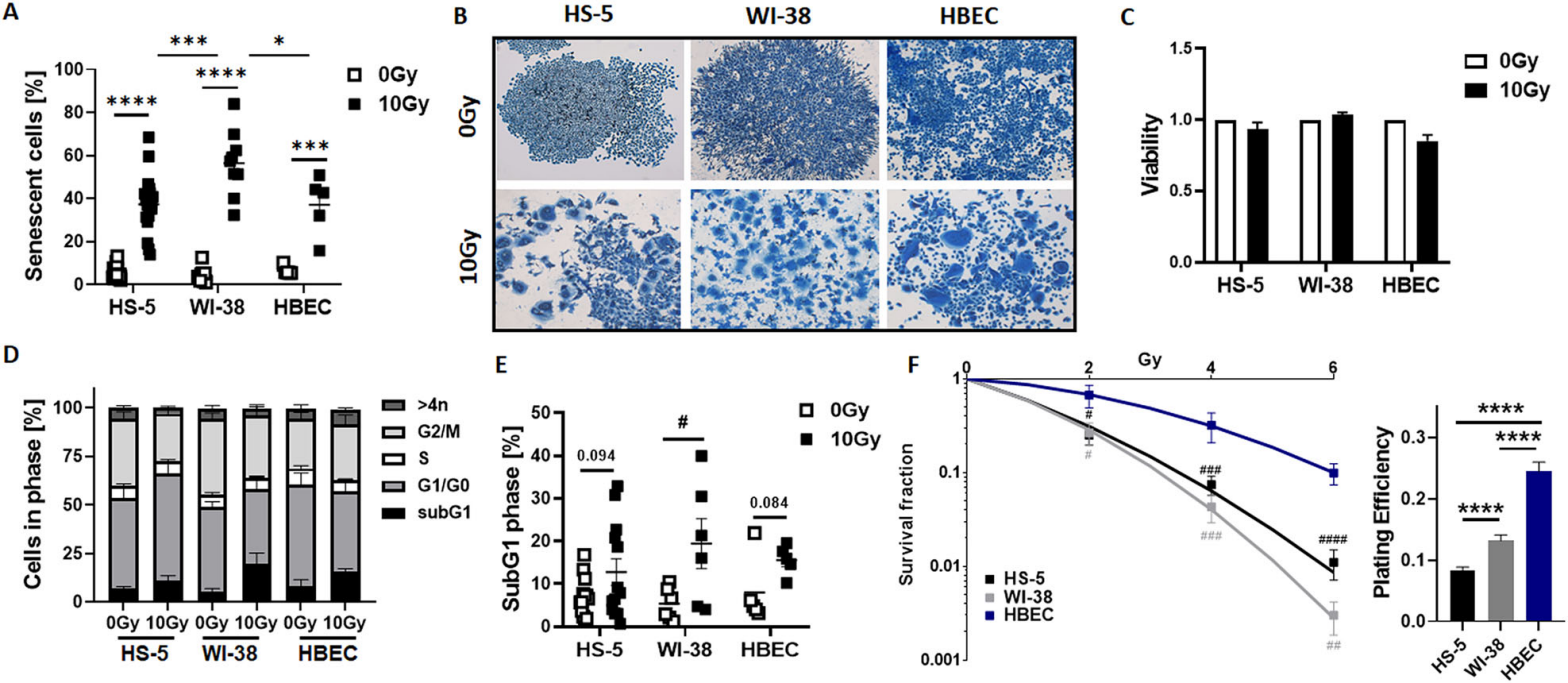
RT-induced senescence in 2D cultured fibroblasts and epithelial cells. **A** RT-induced senescence formation was analyzed by C12FDG staining of 2D cultured fibroblasts (HS-5 and WI-38) and epithelial cells (HBEC) prior flow cytometric analyses at 96 h post treatment with 10 Gy. Graph depict data from 3 to 5 independent experiments. Individual symbols represent different biological replicates. *P* by two-way ANOVA, followed by post hoc Sidak’s multiple comparisons test: *p ≤ 0.05, ***p ≤ 0.005, ****p ≤ 0.001. **B** Morphological alterations in response to RT treatment were visualized following crystal violet staining. Representative photographs from control (0 Gy) and RT (10 Gy)-treated cells are exemplarily shown. Magnification: 5×. **C** Viabilities of indicated cells were analyzed with or without radiation treatment (10 Gy) after 96 h using the WST-1 reagent. Estimated values were related to respective control treatment (0 Gy; set as 1). Data represent mean values ± SEM from 4 to 5 independent experiments measured in quadruplets each. **D** Cell cycle phases were analyzed by flow cytometry. Graphs consist of data from 4 to 5 individual experiments (with SEM). **E** Apoptotic (subG1) cell fractions were additionally depicted. *P* by unpaired (two-tailed) *t* test depicted as #*p* ≤ 0.05. **F** Clonogenic survival of respective cells was evaluated following RT with indicated doses (0–6 Gy) at 10 days post treatment. Data show the surviving fractions (left) and the plating efficiencies (PE at 0 Gy, right) from 3 to 4 independent experiments measured in triplicates each (means ± SD). *P* by unpaired (two-tailed) *t*-test depicted as #*p* ≤ 0.05, ##*p* ≤ 0.01, ####*p* ≤ 0.001 (compared to HBEC) and by one-way ANOVA, followed by post hoc Tukey’s multiple comparisons test: ****p ≤ 0.001.

According to previous investigations concerning senescence induction following RT in cultured lung epithelial cells [19], we determined potential RT-induced senescence in cultured fibroblasts (Fig. 1, Supplementary Fig. S2). Similar to human bronchiolar epithelial cells (HBEC; from 6.49 ± 0.93% at 0 Gy to 37.12 ± 3.03% at 10 Gy; mean ± SEM; *n* = 5 biological replicates per group), senescence was induced in HS-5 (from 5.48 ± 0.69% to 37.31 ± 3.03%; *n* > 10) and WI-38 lung fibroblasts (from 5.31 ± 1.16% to 56.46 ± 5.11%; *n* = 9) following RT (96-hour time point) (Fig. 1A), an effect that was dose dependent (Supplementary Fig. S3). Compared to HS-5 fibroblasts, WI-38 fibroblasts showed the highest levels of senescence being significantly higher compared to HS-5 (*p* ≤ 0.005) and HBEC (*p* ≤ 0.05) cells (Fig. 1A). Morphological alterations (Fig. 1B, Supplementary Fig. S2) confirmed potential senescent cell states in all cell types while the cells remained viable (Fig. 1C). The previously reported p21-p53 axis involvement in this induction in lung epithelial cells [19] could be confirmed, while this was not the case for both fibroblasts (Supplementary Figs. S3 and S4). P21 levels following RT were reduced in the HS-5 cells and even not detectable in the WI-38 cells, although loss of LMNB1 as senescence biomarker was prominent in both fibroblasts. Of note, a reduction of the membrane protein CAV1 was detected in both fibroblasts, an effect that previously could be linked to resistance-promoting properties of irradiated fibroblasts [27–29]. However, a reduction of CAV1 levels following RNA interference did not affect the degree of RT-induced senescence in fibroblasts (Supplementary Fig. S5). Cell cycle alterations revealed reduced cell numbers in the G2/M phases of both fibroblast cell types after RT, but the reductions were not significant (Fig. 1D). Cell death induction via subG1 measurements accounting for RT-induced apoptosis (Fig. 1E) showed significant increases in cell death only for the WI-38 cells (from 5.34 ± 1.56% at 0 Gy to 19.42 ± 5.81% at 10 Gy; mean ± SEM; *n* = 6). In HS-5 fibroblasts (from 7.02 ± 1.23% to 12.77.31 ± 3.06%; *n* = 12; *p* = 0.094) and HBEC (from 7.99 ± 3.51% to 15.55 ± 1.58%; *n* = 5; *p* = 0.088) there was only a trend for increased apoptosis levels at 96 h post RT (Fig. 1D, E). Clonogenic survival analysis further revealed a more resistant phenotype of epithelial cells compared to both fibroblasts (Fig. 1F). These results clearly indicate that senescence is the primary cell fate after irradiation in all these cells.

**Fig. 2:**
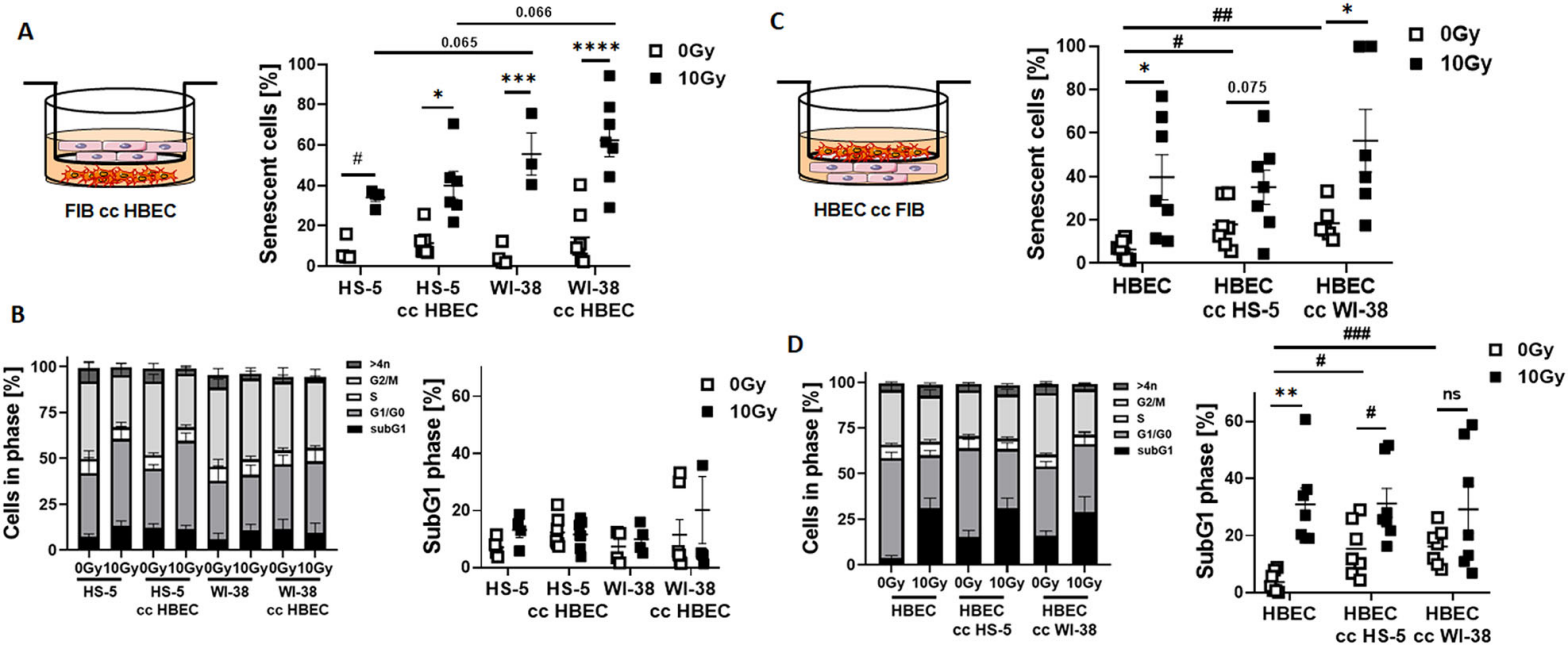
RT-induced senescence of fibroblasts is not affected by co-cultured HBEC cells (transwell co-culture approach). HS-5 were cultured alone (‘empty’ transwell; control condition) or together with HBEC (in indirect co-culture; cc HBEC) for 24 h prior to RT with 0 (Ctrl) or 10 Gy, and analyzed after additional 96 hours (**A**, **B**). **A** Senescence induction of respective HS-5 fibroblasts were analyzed following C12FDG treatment. **B** Cell cycle phases and apoptotic cells (subG1) were analyzed by flow cytometry. In the other way round, HBEC were cultured alone (‘empty’ transwell) or together with HS-5 (in indirect co-culture; cc HS-5) for 24 h prior to RT with and analyzed after 96 hours post RT (**C**, **D**). **C** Senescence, **D** cell cycle phases and apoptotic cells were determined. Individual symbols represent different biological replicates. *P* by two-way ANOVA, followed by post hoc Tukey’s multiple comparisons test: *p ≤ 0.05, **p ≤ 0.01, ***p ≤ 0.005 ****p ≤ 0.001 and additionally by unpaired (two-tailed) *t* tests depicted as #*p* ≤ 0.05, ##*p* ≤ 0.01, ###*p* ≤ 0.005.

Next, we had a closer look at potential interacting processes, e.g., if senescent epithelial cells affect RT-induced senescence or RT-induced-apoptosis in fibroblasts or the other way round. We therefore repeated experiments in a more direct approach using transwell co-culture systems (Fig. 2). Concerning senescence induction, the co-culture of fibroblasts with HBEC cells did not really affect senescence levels of both fibroblasts types following RT [HS-5: from 7.55 ± 2.82% at 0 Gy to 34.13 ± 2.07% at 10 Gy; mean ± SEM at control conditions (empty transwell; *n* = 4) and from 11.46 ± 2.59% to 40.07 ± 6.94%; *n* = 7 upon HBEC co-culture; WI-38: from 4.99 ± 2.52% to 55.58 ± 10.46% at control conditions (*n* = 4) and from 14.28 ± 5.20% to 62.40 ± 8.18% upon HBEC co-culture (*n* = 7)] (Fig. 2A). Even so, cell cycle distributions were not really affected by the co-culture, and not apoptosis induction as estimated by the lack of induced subG1 fractions (Fig. 2B). Even a lack of subG1 induction was noticed for the WI-38 cells when cultured under control conditions (empty transwells), which is in contrast to the observed subG1 levels following RT of classically 2D cultured WI-38 cells (Fig. 1E), but could be due to the medium alterations used in this transwell setup (fibroblast/epithelial medium, ratio 1/1). The degree of RT-induced senescence induction in HBEC cells was not affected by co-cultured fibroblasts [from 6.34 ± 1.51% to 39.70 ± 10.37% at control conditions (empty transwell; *n* = 7) and from 17.84 ± 3.99% to 35.08 ± 7.88%; *n* = 7 upon HS-5 co-culture and from 18.46 ± 3.29% to 56.47 ± 14.39%; *n* = 6 upon WI-38 co-culture] (Fig. 2C). Surprisingly, upon fibroblast co-culture senescence induction was already prominent at non-irradiated conditions (0 Gy; HS-5 co-culture: *p* ≤ 0.05 and Wi-38 co-culture: *p* ≤ 0.01). Similarly, RT-induced apoptosis levels did not differ between in HBEC cells under control and fibroblast co-culture conditions, but upon fibroblast co-culture apoptosis levels were increased already at non-irradiated conditions [from 3.71 ± 1.39% to 30.90 ± 5.63% at empty transwell conditions; *n* = 7, from 15.25 ± 3.60%; *n* = 7 (*p* ≤ 0.05 compared to HBEC Ctrl 0 Gy) to 31.18 ± 5.31%; *n* = 7 upon HS-5 transwell co-culture and from 16.15 ± 2.47% (*p* ≤ 0.005 compared to HBEC Ctrl 0 Gy) to 29.11 ± 8.23% upon WI-38 transwell co-culture] (Fig. 2D). Declining CAV1 levels in HS-5 fibroblasts did not affect RT-induced senescence nor apoptosis induction in the fibroblasts or in the HBEC cells upon transwell co-culture, but senescence and apoptosis inductions in HBEC cells were also increased at non-irradiated conditions when CAV1-silenced fibroblasts were co-cultured in transwells (Supplementary Fig. S6).

From the results so far, we had the impression that all cells cultured in the traditional 2D conditions became senescent upon irradiation, and that cell death induction played only a subordinate role. A more complex culture or namely the co-culture of fibroblasts and epithelial cells using transwells showed a similar picture for both fibroblast types: the cells became senescent upon irradiation and the co-culture of epithelial cells had no (significant) effect on the fibroblasts. Vice versa, co-cultured fibroblasts did not really affect RT-induced epithelial senescence but increased already basal epithelial senescence levels. Similarly, apoptosis levels were increased. Thus, the cells seem to influence each other, particularly fibroblasts impacted here on adjacent epithelial cell fates. Therefore, we wanted to achieve more direct, in vivo*-like* conditions the respective cell co-cultures.

**Fig. 3:**
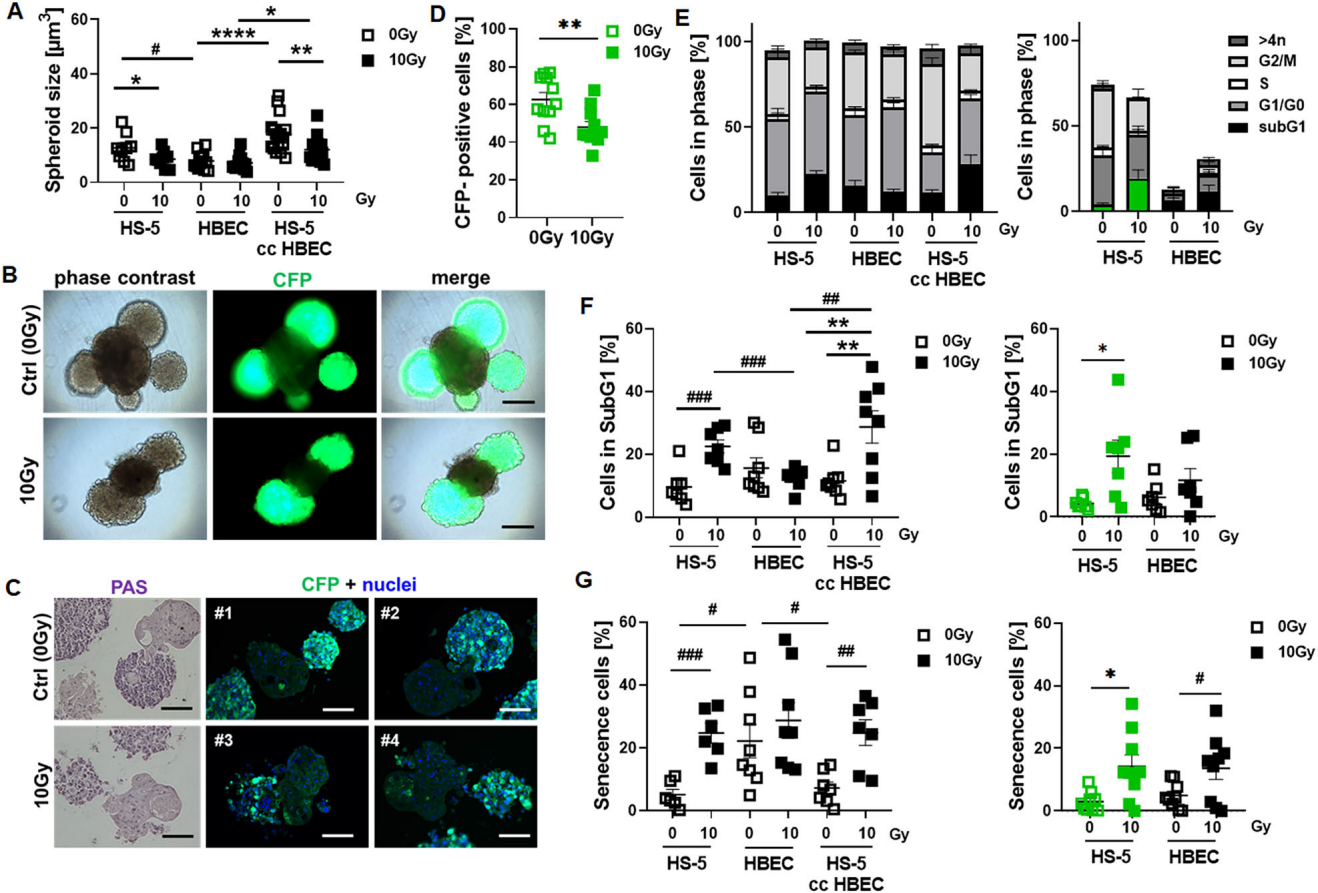
Radiation-induced senescence in fibroblasts promotes senescence of epithelial cells in direct spheroidal co-cultures. HS-5 and HBEC cells were cultured as spheroids either alone (2000 cells per spheroid) or together (HS-5 cc HBEC; 2000 HBEC cells together with 250 HS-5 fibroblasts per spheroid). **A** Spheroid growth was measured 96 hours post RT with 10 Gy and respective volumes were calculated. Graphs depict the measurements from 5 to 8 independent experiments where at least 4–8 spheroids per condition each were measured. **B** Representative live cell pictures of plated HBEC cells (2000 cells/well) together with HS5 fibroblasts (250 cells per well) labeled in green (cyan-fluorescent protein, CFP) at the d11 time point are shown. Magnification 20×. Scale bar represents 100 µm. **C** Periodic acid–Schiff (PAS) staining on paraffin-embedded spheroids was used for histological evaluation (11-day time point). Scale bar represents 150 µm. Immunofluorescent analysis was performed in order to visualize epithelial cells interacting with fibroblasts (green). Representative pictures are shown. Hoechst was used for nuclei staining. Magnification 20×. Scale bar represents 150 µm. # indicate different spheroids (different biological replicates). **D** The number of CFP-expressing cells (fibroblasts, FIB) in whole spheroids was determined by flow cytometry (96 h post RT) following generation of single cell suspension. Individual symbols represent different biological replicates. *P* by unpaired (two-tailed) *t*-test depicted as ##*p* ≤ 0.05. **E** Distribution of cell cycle phases and apoptotic cells (**F**) were determined in whole spheroids (left panels). The significant differences in the G1/G0 and G2/M phases (*p* ≤ 0.005 and *p* ≤ 0.001 respectively) for HBEC-HS-5 spheroids upon RT are not indicated. Respective signals were additionally related to the CFP-expressing fibroblasts (indicated by green color). The significant difference in the G2/M phases (*p* ≤ 0.01) for CFP-expressing HS-5 cells within the HBEC-HS-5 spheroids upon RT is not indicated. **G** Induced senescence levels of respective cultures were analyzed 96 h post RT with 10 Gy following DDAOG treatment. *P* by two-way ANOVA followed by post hoc Tukey’s multiple comparisons test: *p ≤ 0.05, **p ≤ 0.01, ****p ≤ 0.001 and additionally by unpaired (two-tailed) *t* tests depicted as #*p* ≤ 0.05, ##*p* ≤ 0.01 and ###*p* ≤ 0.005 (**A**, **F**, **G**).

We previously established a simple protocol for the spheroidal co-culture of normal epithelial cells together with fibroblasts in a basement matrix-free manner using ultra-low attachment plates [30]. We next used this system to investigate the differential RT response of both cell types in 3D and thus in an in vivo-like manner, either in individual cell type cultures or together in fibroblast-epithelial co-cultures interacting directly (Fig. 3, Supplementary Movie 1). Concerning the spheroid growth, it was noted that fibroblasts cultured alone showed biggest spheroid sizes, and fibroblasts (FIB) were able to increase the spheroid growth in respective HBEC-FIB spheroidal co-cultures compared to HBEC spheroids cultured alone. This might already indicate an increased proliferation potential of the FIB compared to HBEC, with the latter one more differentiating than proliferating (Fig. 3A, Supplementary Movie 2). Growth retardation in response to RT was only prominent in spheroids when fibroblasts were cultured alone or in combination with HBEC cells indicating already a certain degree of resistance of spheroidal cultured HBEC cells. The growth of single HS-5 fibroblast spheroids significantly decreased from 12.72 ± 1.68 µm^3^ (mean ± SEM) at 0 Gy to 8.58 ± 0.99 µm^3^ at 10 Gy 96 h post RT (*n* = 9 per condition), remained stable for single HBEC spheroids (7.96 ± 0.83 µm^3^ at 0 Gy and 7.17 ± 0.74 µm^3^ at 10 Gy; *n* = 13), and also significantly decreased for HBEC-HS-5 spheroids (from 17.71 ± 1.59 µm^3^ at 0 Gy to 12.04 ± 1.05 µm^3^ at 10 Gy 96 hours post RT; *n* = 17). Live cell imaging, histologic evaluations and respective flow cytometry analyses confirmed the reduction of (CFP-labeled) fibroblast numbers following RT (Fig. 3B–D) from 62.63 ± 7.70 (0 Gy) to 47.99 ± 2.93 (10 Gy; *n* = 11) (Fig. 3D). Cell cycle analyses and in particular, estimation of subG1 levels as apoptotic characteristics confirmed a significant induction of cell death in FIB and in FIB-HBEC spheroids in response to RT [from 9.73 ± 2.09 (0 Gy) to 22.54 ± 2.01 (10 Gy; *n* = 7) for HS-5 and from 11.63 ± 2.09 (0 Gy) to 28.67 ± 5.14 (10 Gy; *n* = 8) for HBEC-HS-5 spheroids], both at the expense of cells in the G2/M phase, while a respective response in HBEC cells [15.73 ± 3.01 (0 Gy) and 12.59 ± 1.09 (10 Gy; *n* = 8)] was lacking (Fig. 1E, F). If one now looks at the signal of the co-culture differentially, since the FIB are marked in green, one could see that the FIB in the spheroidal co-cultures were significantly more sensitive with regard to cell death induction [from 4.30 ± 0.68 (0 Gy) to 19.27 ± 5.10 (10 Gy; *n* = 7)] than the HBEC cells, which did not show apoptosis induction [6.27 ± 1.77 (0 Gy) and 11.74 ± 3.74 (10 Gy; *n* = 7)]. A slightly different picture emerged with regard to radiation-induced senescence. A significant induction of senescence was found in the FIB and HBEC-FIB spheroids [from 5.10 ± 1.72 (0 Gy) to 24.70 ± 3.16 (10 Gy; *n* = 6) for HS-5 and from 7.20 ± 1.99 (0 Gy) to 24.85 ± 4.09 (10 Gy; *n* = 7) for HBEC-HS-5 spheroids], while no induction was found in the pure HBEC spheroids [(22.19 ± 5.31 (0 Gy) and 28.74 ± 5.70 (10 Gy; *n* = 7)], although basal senescence levels were already higher in pure HBEC spheroids under non-irradiated conditions (Fig. 3G). A differential analysis of this result - again relative to the GFP signal - showed that the extent of senescence induction in the co-cultures was equally (significantly) distributed between FIB and HBEC [from 2.97 ± 1.04 (0 Gy) to 14.24 ± 3.68 (10 Gy; *n* = 7) for CFP expressing HS-5 and from 4.96 ± 1.38 (0 Gy) to 13.64 ± 3.54 (10 Gy; *n* = 7) for the co-cultured epithelial cells]. Similar to the 2D results (Supplementary Fig. S4) no induction of the senescence marker CDKN1A/ p21 could by observed in the fibroblasts of HBEC-HS-5 spheroidal co-cultures; and surprisingly an induction within the epithelial cells could be hardly seen, at least not via immunohistochemistry whereas loss of LMNB1 was prominent in both cell types (Supplementary Fig. S7). Phosphorylated NF-κB p65 was expressed particularly in the fibroblasts, with already high immunoreactivities in non-irradiated conditions, and a decline in CAV1 could be observed here.

Thus, the induction of senescence in the HBEC cells in the co-cultures appears to be favored and/or caused by the induced senescence in the FIB, since no senescence induction could be detected in the pure HBEC spheroids. This observation was confirmed using WI-38-HBEC spheroids (Supplementary Fig. S8). It can be noted that in the in vivo-like spheroidal culture of the cells - especially with regard to the epithelial cells here - a different picture emerges than in the 2D cultivation (Supplementary Fig. S9). Simply put, in the 2D cell cultures, all cells appear to become senescent during the course of RT and apoptosis induction appears to play only a minor role. Significantly increased apoptosis levels could only be detected for Wi-38 fibroblasts. Upon more complex cell culture conditions, using either transwells or direct spheroidal epithelial-fibroblasts, the fibroblasts seem to affect more the epithelial cells than the other way round. Pure HBEC spheroids are quite resistant to RT where no senescence nor apoptosis induction could be detected. The (more proliferating) fibroblasts undergo apoptosis and senescence in pure spheroids and so upon co-culture with HBEC in respective spheroids. In the latter, even HBEC cells become senescent after RT. Thus, fibroblasts promote senescence in neighboring epithelial cells, although the mechanism remains unclear, as do relevant SASP factors that could promote senescence in epithelial cells in a paracrine manner from senescent fibroblasts.

Concerning the potential impact of differential CAV1 levels in fibroblasts, levels which decline in fibroblast following RT as shown in Supplementary Fig. S3 and as previously reported [27–29], respective investigation using CAV1-silenced fibroblasts in our 3D spheroid model revealed significantly increased senescence levels when CAV1-silenced fibroblasts were cultured alone as spheroids (Supplementary Fig. S10). Spheroidal growth in contrast was increased following CAV1 silencing. In fibroblast-epithelial co-cultures however, no impact of the differential CAV1-levels is fibroblasts could be observed. The number of both fibroblast variants decreased equally in the 3D cultures following RT, as indicated by the reduced GFP signals, which was accompanied by increased cell death induction (increased subG1 values), but the extent of senescence was not altered, neither in fibroblasts nor in epithelial cells in these co-cultures. Thus, RT induced a decline of CAV1 in fibroblasts and RT induced senescence in the remaining fibroblasts that did not undergo apoptosis. However, a reduction of CAV1 seems not to be sufficient to foster RT-induced senescence at least not in more complex in vivo-like spheroidal epithelial-fibroblasts co-cultures.

**Fig. 4:**
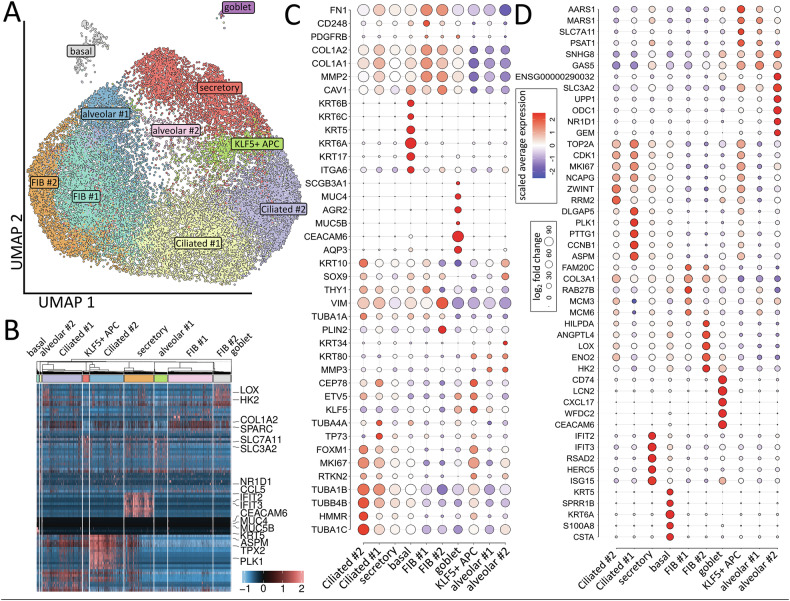
Composition of generated lung spheroids. For scRNA-seq analysis, harvested epithelial-fibroblast-spheroids (after a 11-days culture) were dissociated, and single cells were subjected for bead-based mRNA isolation, bead retrieval and subsequent whole transcriptome analysis. All further analyses were conducted with R packages. **A** Uniform Manifold Approximation and Projection for Dimension Reduction (UMAP) of all single cells (28.587 in total) colored by identified clusters following single cell RNA sequencing analysis. **B** Row-normalized heat map of top ten differentially expressed genes per cluster. Important genes are indicated by name. Cell-type labels for each cluster are based on expression of canonical cell-type markers (**C**) and the top 5 expressed genes per cluster (**D**) displayed in the dot plot. Size of the dots indicate the percentage of cells in which this gene was found, the color indicates the normalized value of the expression.

Single cell RNA-sequencing (scRNA-seq) of co-cultured FIB-HBEC spheroids was then performed in order to proof presence of the different epithelial and mesodermal lung cell subsets in our system and to characterize respective phenotypes in more detail (Fig. 4). Clustering of the single cells revealed the presence of 10 different main cellular subsets (Fig. 4A, Supplementary Table S1). Among the different subsets, basal cells expressing KRT5, KRT6A/B/C and ITGA6 were identified (Fig. 4B, C). Two TUBA (TUBA1A/B/C) and TUBB4B, TUBA1C and TUBB2B expressing ciliated (#1, #2) cell clusters were distinguished, with cluster ciliated #1 (ASPM, PLK1, TP73, CEP78) comprising (differentiating) luminal epithelial cells undergoing ciliogenesis and with cluster ciliated #2 comprising more mature ciliated cells (at all higher TUBA1A/B/C levels, KRT10, ZWINT). Secretory cells and particular goblet cells were identified by the expression of ARG2, MUC4/5B, AQP3 and CEACAM6 close to designated secretory epithelial cell cluster with potential immune conferring capabilities expressing ISG15, IFIT2/3 and HERC5. Three alveolar and two fibroblastic clusters were further identified. Cluster KLF5 + APC (KLF5-positive alveolar progenitor cells) consists of (proliferating) AECI/II lineage-prone cells (AECI/II-differentiating BASC cells) expressing KLF5, ETV5, FOXM1, and SLC7A11. More advanced differentiated alveolar cells were designated based on the additional expression of SLC3A2, MMP3, KRT34 (alveolar #2) as AT2-like (or transitional AT2) epithelial cells, beside AT1-like (alveolar #1) alveolar epithelial cells expressing increased MARS1, AARS1, and GAS5 levels close to the alveolar fibroblasts cluster (FIB#2). Beside classical mesodermal gene (FN1, COL1A1/2, MMP2) expressed by both fibroblastic clusters, the adventitial FIB #1 clusters was characterized by the additional expression of CD248, THY1 and RAB27B and thus more reactive ECM-remodeling related genes, while additional expressed genes in the alveolar FIB#2 cluster complied VIM, PLIN2, CAV1 and more metabolism-related genes particularly LOX, ENO2, HK2. Collectively, scRNA-seq revealed the presence of classical epithelial and mesenchymal subpopulations of lung cells in more complex lung structures following direct spheroidal cultivation of both commercially available normal bronchial epithelial cells together with fibroblasts, which further highlights epithelial cell-fibroblast spheroids as a physiologically relevant 3D lung model.

**Fig. 5:**
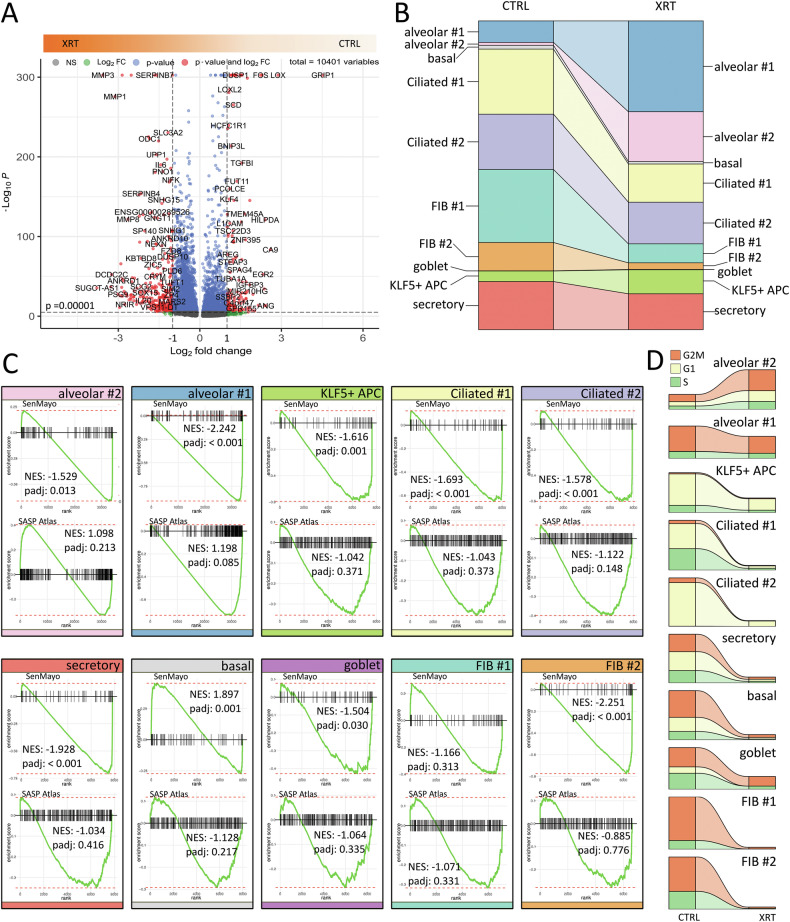
Radiation induced changes in lung spheroids. **A** Volcano plot of all differentially expressed genes (cluster-independent) obtained from scRNA-seq data sets. Labels are added to genes, which have an absolute log2 fold-change higher than 1 and a *p*-value lower than 0.00001. **B** Changes of cluster sizes due to irradiation. **C** Enrichment plots of a gene set enrichment analysis of the SenMayo and the SASP Atlas gene sets for each cluster. *P*-values are calculated by adaptive multi-level split Monte-Carlo scheme (fgsea R-package). **D** Cell cycle classification per cluster before and after RT.

We then of course investigated the radiation response in more detail within our FIB-HBEC spheroidal co-cultures to further gain insight into the cell-type specific molecular alterations following RT and particular with respect to single cellular fates by performing (again) scRNA-seq of RT-treated spheroids (Fig. 5). A volcano plot analysis was used to highlight differential gene expressions in following RT (Fig. 5A, Supplementary Table S2). Using the log-fold change >1.5 and the adjusted *p*-value of <0.001 167 transcripts were found to be differentially expressed in both cell states with 126 genes found to be upregulated in irradiated spheroids compared to 41 upregulated genes in control (non-irradiated) cultures. Changes of cluster sizes due to RT revealed increases in portion of cells of the alveolar #1 (AT1-like epithelial cells), alveolar #2 (AT2-like or transitional AT2 cells) clusters, and KLF + APC, while the (small) portion of the basal cell cluster remained unchanged (Fig. 5B). The respective airway clusters (Ciliated #1 and #2 as well as the small cluster of goblet cells) were slightly reduced, and decrease was also estimated for the alveolar FIB #2 cells, while the portion of adventitial FIB #1 cells decreased more. Concerning the cellular fate in response to RT, and particularly with respect to RT-induced senescence, the molecular alterations were further investigated. In order to validate that RT-treated epithelial-fibroblastic spheroids displayed a senescent gene expression signature, and particularly which cells inside this cellular assembly, we performed gene set enrichment analysis (GSEA) using well-known senescent phenotype genes reported by others (Fig. 5C). Using the “SenMayo” gene set allowing identification of senescent cells and prediction of senescence-associated pathways across tissues [31], an enrichment of genes identifying senescence could be estimated for all clusters except for the basal and the adventitial FIB #1 clusters. Concerning the SASP atlas, a gene set for secretory SASP factors exclusive to ionizing radiation as previously described [32], a trend for enrichment could be observed in all clusters except for the alveolar #2 cluster, indicating a generally altered secretion profile of the spheroids following RT.

In order to eventually unravel the mechanism behind the potential senescence induction, cell cycle classification per cluster before and after irradiation was performed together with gene expression analysis of cell cycle and senescence-associated genes as well as of apoptosis-related genes as revealed from the single cell RNA-seq data sets (Fig. 5D and Supplementary Fig. S11). According to the reported increase of the alveolar #2 clusters increases in G2/M phases could be detected, while G2/M phases remained nearly stable (slightly decreasing) in the alveolar #1 cluster, together with decreasing G1 phases while lacking obvious G2/M phases in the KLF5 + APC cluster (Fig. 5D). Decreases of the remaining portions came along with decreases in all cell cycle phases, particularly for the G2/M phases within the airway (basal, goblet and secretory) and fibroblastic clusters. Like the KLF5 + APC cluster, the ciliated clusters were generally characterized by cells in the G1/G0 phase, which portions decrease following RT. Surprisingly, no cluster showed a clear increase in CDKN1A levels following RT, at least not on mRNA levels (Supplementary Fig. S11), which is consistent with the immunohistochemical investigation (Supplementary Fig. S7). Induced expression levels of the senescence-related gene CCND1 became prominent in both FIB clusters, the alveolar #1 cluster and to a lesser extent in the ciliated #2 and secretory cluster. Similarly, MAPK14 encoding p38α mitogen-activated protein kinase (MAPK), the prototypic member of the p38 MAPK family (and generally known to be induced in response to cellular stress) was induced here in response to RT in the adventitial FIB #1 and goblet clusters, and to a lesser extent in the alveolar FIB #2 and secretory clusters. Together with the results stated above, this might indicate that the RT response and the induction of both apoptosis and premature senescence in fibroblasts might be due to p38α activation. Thus, based on RT-induced gene alterations, together with the significantly enriched genes of the ‘SenMayo’ signature and the cell cycle-related changes, RT-induced senescence could be stated for the airway clusters, particularly for the (remaining) secretory and goblet cells and for both FIB clusters. The three alveolar-related clusters showed also these senescence characteristics but increase in numbers, a fact that might be indicative for regenerative processes, together with the basal cluster that lacks a clear senescent phenotype.

**Fig. 6:**
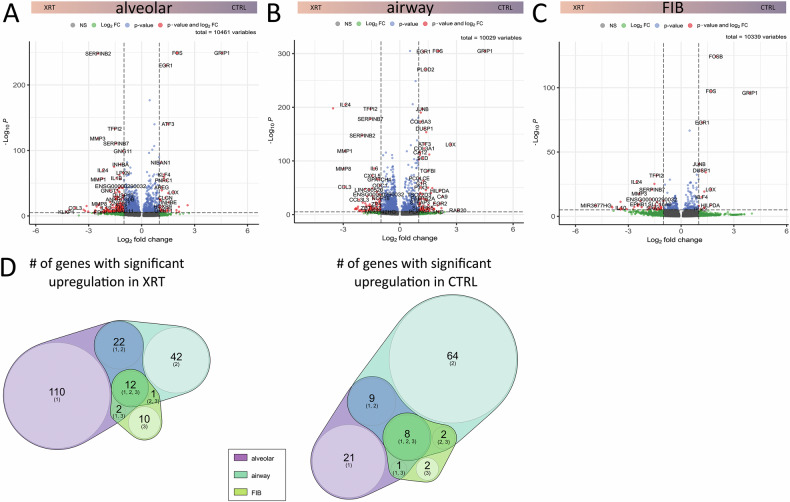
Potential RT-induced signatures in lung spheroid meta clusters. Volcano plot of all genes in **A** alveolar (comprising the alveolar clusters #1, #2, and KLF5 + APC), **B** airway (Ciliated #1, #2, secretory, goblet and basal) and **C** FIB (#1 and #2) meta cluster. Labels are added to genes, which have an absolute log2 fold-change higher than 1 and a *p*-value lower than 0.00001 (Mann–Whitney *U* test). Venn diagrams of significantly up regulated genes in Ctrl and RT-treated (XRT) lung spheroid meta clusters.

**Fig. 7:**
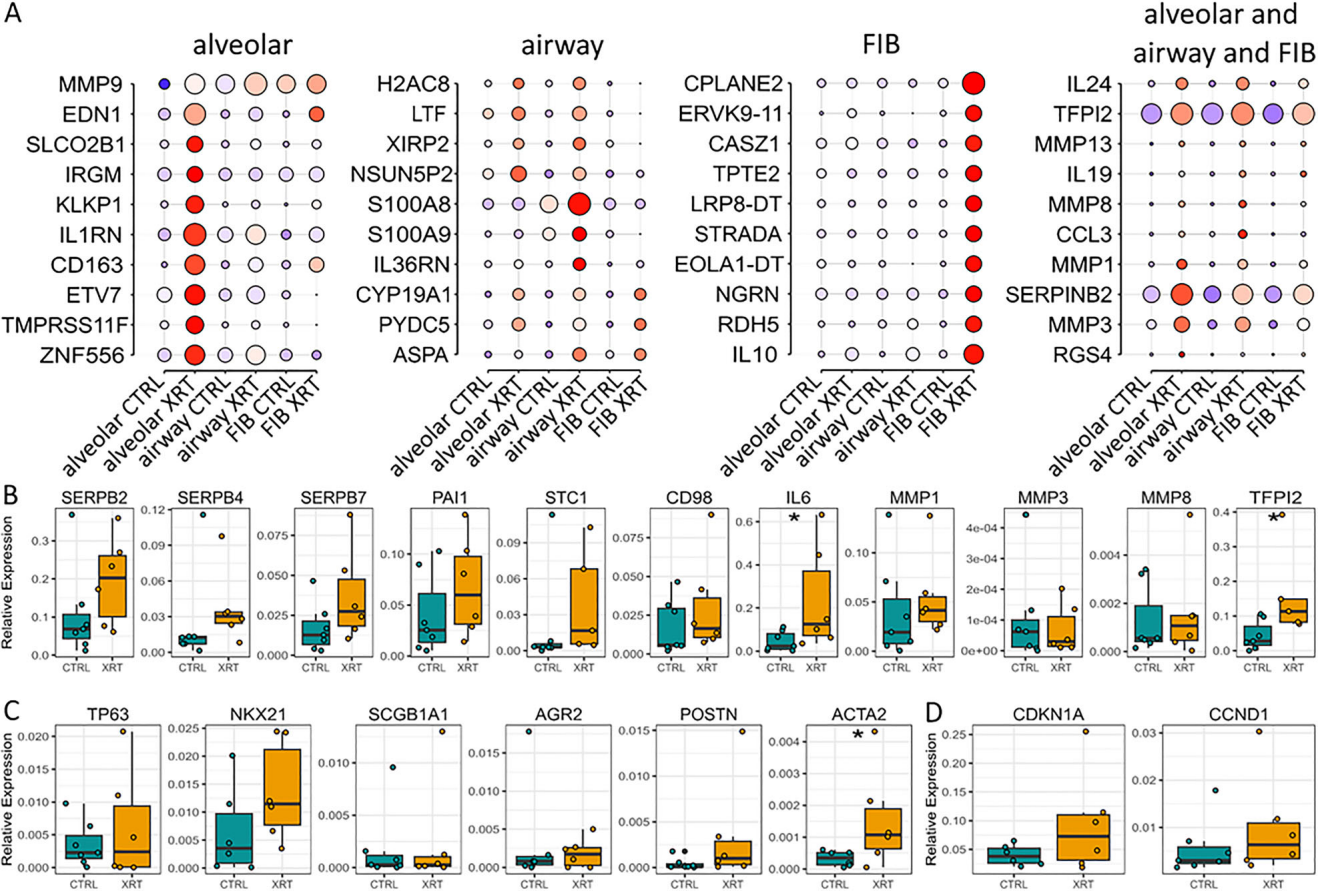
Expression of significant genes in alveolar, airway and FIB meta cluster. **A** Dot plot of the top differentially expressed genes per meta cluster before and after irradiation. Size of the dots indicate the percentage of cells in which this gene was found, the color indicates the normalized value of the expression. Indicated transcript levels of known SASP factors (**B**), lung maker genes (**C**) and of the senescence marker CDKN1A and CCND1 (**D**) were quantified using Real-Time RT-PCR and are shown as relative expression to beta-actin. Data are shown as interquartile range. Individual symbols indicate different biological replicates (*n* = 5–6 biological replicates per gene and condition). Mann–Whitney *U* test. *0.05.

Based on these results we finally investigated the molecular changes concerning the alveolar (#1/2, and KLF + APC), airway (basal, ciliated #1/2, secretory, and goblet cells) and FIB (#1/2) meta clusters in order to unravel and highlight RT-induced changes that could be used to understand the alterations of RT-induced lung injury (Fig. 6, Supplementary Fig. S11). Volcano plot analysis visualized differentially expressed genes in meta clusters (Fig. 6A–C). Using the log-fold change > 1 and the adjusted *p*-value of <0.01, 185 transcripts were found to be differentially expressed in three alveolar clusters, with 146 genes found to be upregulated in response to RT compared to 39 upregulated genes in Ctrl (non-irradiated) cells (Fig. 6A). 160 transcripts were found to be differentially expressed in five airway clusters, with 77 genes found to be upregulated in response to RT compared to 83 upregulated genes in cells under Ctrl conditions (Fig. 6B). 38 transcripts were found to be differentially expressed in both FIB clusters, with 47 genes found to be upregulated in response to RT compared to 13 upregulated genes in non-irradiated cells (Fig. 6C). Comparing the gene alterations concerning the meta clusters following RT revealed differentially expressed genes in the alveolar clusters comprising 110 genes upregulated following RT compared to 42 upregulated genes in the airway and 10 upregulated genes in the irradiated fibroblast subsets (Figs. 6D and 7 and Supplementary Table S3). An intersecting gene signature consisting of 12 genes was found to be induced in the epithelial-fibroblast spheroids after irradiation (SERPINB2, SERPINB4, SERPINB7, TFPI2, MMP1, MMP3, IL24, BEX1, SLC16A6, SNHG4, CEMIP, and the F-Box Only Protein 25 Pseudogene (ENSG00000290032)), which could be used as additional as well as lung cell type-specific senescence gene sets. No alterations could be observed for CAV1 expression levels (not shown).

## Discussion

In the context of chronic lung diseases, lung cancer, and especially RT-induced normal tissue toxicity, several cell types in the lung have been reported to undergo cellular senescence. For example, in pulmonary fibrosis, AT2 epithelial cells and lung fibroblasts were identified as senescent in both animal models and patients and the respective SASP played a central role in driving fibrosis through the secretion of pro-inflammatory and pro-fibrotic mediators [33–35]. For the epithelial cells it was shown that accelerated mitochondrial damage [36] and increased WNT/β-catenin signaling [37] in the course of pulmonary fibrosis led to increased senescence and reprogramming in AT2 cells. The increased WNT/β-catenin signaling even impaired progenitor cell function and hampered lung repair, thereby perpetuating tissue damage [37]. For RT-induced senescence in AT2 cells it was further shown that elevated reactive oxygen species (ROS) levels, generated through NADPH oxidase activation, stimulated fibroblast proliferation and collagen deposition, which exacerbates pulmonary fibrosis [38]. The SASP of senescent fibroblasts in turn has been shown to exert paracrine fibrogenic effects on adjacent healthy fibroblasts, inducing the expression of ACTA2 thereby driving differentiation into myofibroblasts [39]. This effect, however, was not observed for senescent bronchiolar epithelial cells, indicating a cell type-specific contribution to RILI [40]. Senescence of bronchial epithelial cells following RT-induced cellular stress however was suggested as a central process for the initiation and progression of pneumonitis and fibrosis [19, 21, 23, 41]. Limiting RT-induced senescence and SASP expression levels in these cells limited associated side effects particularly vascular dysfunctions and inflammation early after irradiation (acute effects) [19]. Similarly, abrogation of certain aspects of these cells, namely signaling inhibition of the SASP-factor Ccl2/Mcp1 mediated radioprotection [21].

Although these studies were predominantly based on animal experiments, i.e., murine models of RT-induced pneumopathy, it is clear that RT-induced senescence is an important, if not central, process for the initiation and progression of RILI. However, it remains unclear how and when which cells in the lung become senescent during thoracic irradiation, and how these cells then interact during the course of the disease. Here we investigated radiation-induced senescence of fibroblasts and epithelial cells in order to specify possible interactions that could presumably improve the understanding of the clinical picture of RILI, and could also serve as potential prognostic and/or therapeutic targets. First, we observed that senescence is induced in all the cell-types used following RT when the cells were classically cultivated in 2D. At the same time, induced cell death levels and particularly induced apoptosis levels were rather low. At the same time, declining CAV1 levels in fibroblasts were observed following RT confirming previous findings [27, 28], while levels in epithelial cells remained unaffected. Due to its various functions CAV1 was shown to contribute to the progress of vascular dysfunction, inflammation and alveolar leakage in the initial stage of acute lung injury (ALI) [42], which was confirmed by the fact that CAV1 knockdown inhibited the occurrence of ALI [43]. Different murine studies were able to show that CAV1 is downregulated in normal lung tissue in the course of RT-induced [44] or bleomycin-induced fibrosis [45]. Particularly within the latter model it was shown, that CAV1 levels in fibroblasts decrease [46]. A decrease of CAV1 levels in fibroblasts was even shown in pulmonary fibrosis patient samples [47]. In previous work we investigated the in impact of CAV1-deficiency especially in fibroblast within the RT response of solid tumors; and revealed that declining CAV1 levels in response to RT caused a resistance-promoting phenotype of the fibroblasts fueling adjacent cancer cells with high energy metabolites and resistance factors, although senescence was not investigated at this point [27–29]. In the present study, we could not clearly link the declining CAV1 levels observed in fibroblasts following RT to senescence induction at least not in classical 2D and not in more complex in vivo-like spheroidal epithelial-fibroblasts co-cultures. In contrast, a downregulation of CAV1 was already linked to premature senescence induction in fibroblasts, which correlated with mitochondrial dysfunctions [48, 49]. CAV1 in fibroblasts may be involved in mediating cellular senescence through linking induced oxidative stress to senescence development by ATM activation, a gene controlling repair of DNA double-strand breaks, and both, p53 and p21 protein upregulation expression following induced DNA damage [48, 50, 51]. Nuclear and cytoplasmic localization of CAV1 following oxidative stress coincided with induced premature senescence, an observation that was accompanied by p38α mitogen-activated protein kinase-dependent phosphorylation of CAV1 [51]. Thus, at least in fibroblasts, the pathway of senescence induction might be regulated by CAV1. A p38-dependent phosphorylation of CAV1 has been described, and CAV1 phosphorylation is usually associated with CAV1 internalization. The more cytoplasmic localization, in turn, influences senescence before CAV1 is possibly degraded and thus CAV1 levels are lowered. For bronchial epithelial cells in contrast loss of CAV1 might not bother as these lung structures are characterized by low or neglectable CAV1 levels. Induced CAV1 levels in turn causes bronchiolar epithelial hyperplasia and atypia here and might foster lung cancer development [52]. Of note, Cav1-deficient mice show histopathologic features that are highly reminiscent in the acute respiratory distress syndrome (ARDS), the severe form of ALI, e.g., thickening of the alveolar septae while alveolar spaces appear significantly smaller or even constricted, features that finally impact on dysfunctions of pulmonary gas exchange and overall lung capacity [53–55]. This might point towards other relevant lung cell types. Cellular alterations like increased numbers of VEGFR2 (KDR)-positive endothelial cells and fibroblasts were reported, both cell types that -together with type 1 pneumocytes and mural cells- show an abundance in CAV1 expression levels (and caveolae). In human lung sections from patients with ARDS, reduced endothelial CAV1 expressions, and severe pulmonary vascular remodeling was reported [56]. Since endothelial cells were not investigated in this study, the complexation of the respective spheroids with these cells is an interesting starting point and will be investigated in future studies. Own observations up to now did not show a decline of CAV1 in endothelial cells following RT at least not in 2D cultures [57, 58]. However, further studies are highly desired to unravel the impact of CAV1 alterations following RT and particularly its (cell-type specific) role in the mechanism of senescence induction.

Regarding the mechanism of RT-induced senescence, no involvement of the CDKN1A/p21-p53 axis could be found in fibroblasts, as we have already observed for epithelial cells [19]. In addition to the well-established roles of p53 and pRB in cellular senescence, evidence also suggests that stress-activated mitogen-activated protein kinase (MAPK) cascades converging on c-Jun N-terminal kinases (JNKs) and p38 MAPKs also play an important role in cell fate in senescence [59, 60]. According to the CAV1-related observations as stated above, a rapid activation of p38 MAPK has already been found in human dermal fibroblasts that underwent premature senescence following TNF-alpha treatment [61]. Senescent fibroblasts further on displayed a permanent phosphorylation of p38 MAPK, an observation that was linked to ROS accumulation and an inflammatory and catabolic phenotype. At the same time, p21-deficient fibroblasts were shown to enter senescence similar to wild-type cells that already indicated that p21 is not essential for senescence induction in fibroblasts [62]. It was even found that in human diploid fibroblasts, which express the papillomavirus type 16 oncogene E6 after the immortalization process (as is the case with HS-5 fibroblasts), low p21 levels result from enhanced p53 degradation [63]. A senescent phenotype here was characterized by accumulated non-phosphorylated pRb and p16, already suggesting that senescence are uncoupled in the absence of normal p21 levels [63]. However, the known abbrevation ‘senescent cell-derived inhibitor 1 (SDI1)’ for p21, a 164 amino acids containing protein with a molecular weight of about 18 kDa already highlights it’s deceisive role in cellular senescence and also aging-related diseases [64–66]. Beside expression regulation by gene transcription, posttranscriptional factors (miRNA and RNA-binding protein), and posttranslational modifications (e.g., phosphorylation and ubiquitylation) finely regulate p21 activity [67, 68]. For example, a caspase-2-mediated (non-enzymatic) mechanism was revealed to regulate p21 expression at the translational level [69]. Posttranslational modifications, such as phosphorylation of several serine and threonine residues and ubiquitination of lysine residues (in the carboxyl-terminal region) in p21, greatly influence p21 activity, e.g., both modifications can lead to the loss of interaction with nuclear PCNA (proliferating cell nuclear antigen); and the latter one mediating p21 proteolysis. Suppression of p21 ubiquitylation and phosphorylation in turn can results in increase stability of the p21 [70, 71]. MDM2 in contrast, a gene found to be induced following RT in complex lung cultures, particularly lung organoids [72, 73], promotes p21 turnover independently of its ubiquitin E3 ligase activity. Therefore, it can be assumed that fibroblasts in particular are unlikely to become senescent via the p21 signaling pathway, at least not in the case of RT, although the exact mechanism here still needs to be elucidated.

In addition to the classical 2D culture methods, we investigated a simple and quite practical method for the direct spheroidal co-cultivation of human bronchial epithelial cells and fibroblasts, which can be considered as an in vivo-like cultivation method [30], especially with regard to the cellular composition of the respective spheroids. We used this model here to specify the RT response of different lung cells. After irradiation, we observed a slight decrease in cilia-bearing and secretory cells in the epithelial fibroblast spheroids, as well as in the inherently scarce goblet cells. With the exception of the basal cells, these airway cells exhibit distinct senescence characteristics. This is consistent with the findings from our own mouse experiments, which showed that the induction of senescence by RT in airway epithelial cells (bronchial epithelial cells) is a central event in thoracic irradiation [19, 23]. The alveolar cells, however, also showed an enrichment of genes that indicate senescence in the SenMayo gene sets. However, the increase in cells after RT rather indicates the regenerative potential of these cells and thus a different cell fate induced by RT, e.g., differentiation into alveolar cells. Thus, the alveolar cells, along with the basal cells, appear to be characterized by a less radiosensitive phenotype. Within the spheroids, a prominent decline in fibroblasts was observed following RT, with adventitial fibroblasts being more affected, while the alveolar fibroblasts showed a clear senescence signature. Similar observations were reported using a stem cell-derived lung model [72]. It has to be noted that the lung cell types formed here in the epithelial-fibroblast spheroids predominantly represent the bronchial epithelium (of the conducting airways), while alveolar cell types are formed less frequently, at least in terms of numbers. This situation could possibly be improved by the intercalation of endothelial cells (potential formation of a blood-blood barrier). Briefly summed up, several features of RILI observed in vivo could be reproduced in vitro. This, in turn, could make the more complex spheroids a useful preclinical model for deciphering the mechanisms of radiation damage and identifying potential biomarkers and drug targets for RILI progression. In addition to cellular losses after RT, the altered secretory phenotypes of the remaining senescent cells lead to the development of a pro-inflammatory and pro-fibrotic phenotype affecting both bronchial epithelia and fibroblasts. Within that scenario, we specify here a gene signature of the interface of alveolar, airway, and FIB metaclusters that is upregulated after RT, which could be used to identify and specify a general and cell-type-independent RT response of the lung, particularly with regard to senescence.

We identified a 12 gene signature that was commonly be induced in irradiated lung spheroids irrespective of the cellular subset. Among these genes certain serpins emerged to be increased (e.g., SERPINB2, SERPINB4, SERPINB7, SERPINE1/PAI-1) together with TFPI2. Serpins are the largest, most broadly distributed and functionally diverse group of (serine) protease inhibitors, which control serine protease activity, including their expression as zymogens (pro-proteases) [74]. SERPINB3 and SERPINB4 for example have been shown to increase mucus production by inducing the transcription factors SPDEF and FOXA3 finally resulting in goblet cell hyperplasia [74, 75]. SERPINE1, also known as plasminogen activator inhibitor-1 (PAI-1), is considered to act as main inhibitor of plasminogen activation due to its regulation of both tissue (tPA) and urokinase plasminogen activator (uPA) and thereby regulating fibrinolysis, but it can even inhibit APC and thrombin in the presence of the cofactors heparin and vitronectin [74]. Increased expression levels of PAI-1 are already known as a characteristic of a number of lung diseases including RILI, and this been closely linked to RT and senescence [23, 76, 77]. As a central part of the SASP, PAI-1 was shown to be overexpressed by senescent (tumor-associated) fibroblasts in the lung following RT and may be involved in the radioresistance of lung tumors [76]. Moreover, PAI-1 turned out to be a critical component in the initiation of cellular senescence [78], e.g., by inducing senescence in lung epithelial via the activation of the p53-p21 axis [79]. This is consistent with our findings. In contrast to the pure epithelial spheroids, which did not develop senescence following RT, spheroids engineered from fibroblasts and epithelial cells showed induced senescence in both the fibroblasts and epithelial cells following RT. Thus, fibroblast-derived PAI-1 could be a relevant SASP factor known to reinforce senescence and/or mediating paracrine senescence in neighboring (epithelial) cells [80, 81], as could the identified additional serpins. Serpins in general and the new identified members here induced following RT could serve as biomarker for RT-induced normal tissue toxicity in general and particularly for RT-induced senescence in lungs. For example, SERPINB2, also known as plasminogen activator inhibitor-2 (PAI-2), is known to be induced following by pro-inflammatory stimuli within lung injury [82, 83]. Of note, SERPINEB2 together with the candidate genes, CDC167, POSTN, and SEC14L1, were suggested to exhibit biomarker potential for airway inflammation [83]. Since increased levels of these inhibitors are generally detected during initial periods of inflammation, it is assumed that serpins are upregulated in order to suppress the inflammatory response [74]. A sustained overexpression of these inhibitors could in turn trigger pro-inflammatory effects. Investigating the modulation of these candidates could lead to new strategies for radioprotection or overall inflammation control.

As an additional candidate, TFPI (tissue factor pathway inhibitor) was identified, and tissue factor (TF) and TFPI were know to play a central role in the process of (acute) lung injury [84]. The transmembrane glycoprotein TF expressed by various tissue cell types, particularly lung epithelial cells plays a central role in (sepsis-associated) blood clotting change via thrombin generation that in turn is controlled by TFPI, which is an endogenous inhibitor of the TF-associated coagulation cascade [85]. TFPI for example is able to reduce the inflammatory responses thereby attenuating pulmonary and systemic coagulopathy [86, 87]. A potential role of tissue factor pathway inhibitor-2 (TFPI2) in cellular senescence was already suggested in aging skin tissues and fibroblasts [88], where TFPI2 might play a regulatory role in the proliferation of different cell types. TFPI2, also called matrix-associated serine protease inhibitor, is a 32 kDa Kunitz-type serine proteinase inhibitor that usually inhibits plasmin and thereby effectively decreases activation of several matrix metallo proteinases (MMPs) namely MMP-1, MMP-3 and MMP-9. These MMPs contribute to the pulmonary inflammatory response within lung injury and can drive lung tissue destruction [89, 90]. TF and TFPI even turned out to be excellent candidate biomarkers for early diagnosis of sepsis and risk stratification in septic patients [91]. Likewise, MMP 3 and MMP9 might serve as biomarkers of severity in COVID-19 patients [92], with pharmacological inhibition of MMP3, also known as stromelysin-1, even displaying a as therapeutic option for ARDS [93]. Expression of the matricellular protease MMP3 by bronchial and alveolar epithelial further contributes to extracellular matrix remodeling as well cell-cell barrier disruption due to its enzymatic proteolysis of junctional proteins (e.g., claudins and occludins) [94].

Conclusively, the studies presented here contribute to a better understanding of RT-induced normal tissue damage. More complex cell culture models should be used, as classic 2D cell cultures - as shown here by the use of lung epithelial cells and fibroblasts - do not adequately reflect RT-induced cell fates observed in vivo. In contrast, the presented results regarding the spheroid model in combination with RT demonstrate that many features of RILI observed in human patients can be reproduced in vitro, making it a useful preclinical model for deciphering the mechanisms of radiation damage and for identifying potential biomarkers and drug targets for RILI. Accordingly, we provide further insights into radiation-induced cell fates of normal lung cells (cell death versus senescence versus regeneration) and provide new candidate gene lists that can be used to identify and specify a general and a cell type-dependent RT response in the lung, particularly with regard to senescence.

## Material and methods

### Cell culture

For normal fibroblast lines HS-5 (CRL-3611) and WI-38 (CCL-75, both from ATCC, Manassas, VA) were cultured in RPMI or MEM199 media (Gibco, Thermo Fisher, Waltham, MA), each supplemented with 10% fetal calf serum (FCS), 100 U/mL penicillin/streptomycin, and 1% non-essential amino acids. Fluorescently labeled and CAV1-silenced HS-5 variants were previously described [27, 28]. The normal lung epithelial cell line HBEC3-KT (CRL-4051–ATCC) was cultured in epithelial cell growth medium (PneumaCult-Ex Basal Medium; Stemcell Technologies, Vancouver, BC). All cell cultures were maintained under standard conditions (37 °C, 5% CO₂ in humidified atmosphere) on standard plastic cell culture dishes (2D) and regularly screened for mycoplasma contamination. For indirect transwell co-culture, distinct cell types were plated independently into 6-well plastic dishes and corresponding transwells (using the respective cell-type dependent media) 24 h prior assembly. Following another 24 h incubation period, the co-culture was irradiated with indicated doses and analyzed at 96 h post RT. Empty transwells cultures (without cells but containing the other cell type-specific media; final fibroblast/epithelial medium, ratio 1/1) were used as control. For 3D spheroidal culture, individual or epithelial-fibroblast cell mixtures were seeded into ultra-low attachment BIOFLOAT 96-well plate (#83.3925.400; SARSTEDT, Nümbrecht, Germany) with 2000 epithelial cells and 250 fibroblast per well in 100 µl PneumaCult-Ex Basal Medium and cultured as previously described [30, 95]. Irradiation treatment of the different cell cultures was carried out using an X-RAD 320 device (Precision X-Ray Inc., North Branford, CT) operating at 320 kV and 10 mA, with a 1.65 mm aluminum filter, at a 50 cm source-to-sample distance, delivering a dose rate of 2.6–2.7 Gy/min at room temperature. For cell viability measurements, WST-1 reagent (#11 644 807 001, Roche/Sigma-Aldrich) was added (1/10) to respective cultures and colorimetric changes were measured at 450 nm (optical density; OD) at the indicated time points as previously described [19, 76].

### Clonogenic survival assay

For clonogenic survival, cells were plated at low densities (100–10,000 cells per well; triplicates) and irradiated with indicated doses [19, 76]. After a 10 days’ culture, the cells were washed with PBS, fixed with 4% (w/v) paraformaldehyde, and subsequently stained with 0.05% Coomassie Brilliant Blue. Colonies (⩾50 cells/colony) were counted, plating efficiencies (number of colonies observed/ number of cells plated) were calculated and survival curves were established by plotting the log of the surviving fraction [number of colonies formed/(number of cells plated × PE)]. Representative plates are exemplarily shown in Supplementary Fig. S1.

### Flow cytometry

Flow cytometry analyses were performed as previously described [72, 76]. For adherent (classically 2D cultured) cells, cells were detached following treatment at indicated time points by trypsinization [30]. Single cell suspensions were generated by re-suspending spheroids in TrypLE for a 15-min incubation (see below) at 37 °C. Digestions were stopped by adding PBS containing 2–5% fetal calf serum (FCS). Cell cycle phases were analyzed using respectively harvested cells in combination with Nicoletti staining solution [50 μg/mL 7-Aminoactinomycin D (7-AAD), 0.1% sodium citrate (w/v), and 0.05% Triton X-100 (v/v) in PBS] (30-min incubation at room temperature prior analyses). Senescence formation was analyzed following bafilomycin A1 (100 nM, Biozol, Eching, Germany) and C12FDG (5-dodecanoylaminofluorescein di-β-D-galactopyranoside; 33 µM) or DDAO galactoside (9H-(1,3-Dichloro-9,9-Dimethylacridin-2-One-7-yl) β-D-Galactopyranoside; 20 µM; both Thermo Fischer Scientific Waltham, MA) incubations as previously described [19, 96]. DDAOG was used for 3D spheroidal co-cultures. Cells were washed twice and subsequently re-suspended in 200 μl FACS buffer (5% FCS in PBS) and analyzed on a CytoFLEX Platform (Beckman Coulter) using the CytExpert Software (Beckman Coulter).

### Western blotting

Generation of whole cell lysates was carried out by scraping cells off into ice-cold RIPA buffer (150 mmol/L NaCl, 1% NP40, 0.5% sodium-deoxycholate, 0.1% sodium-dodecylsulfate, 50 mmol/L Tris/HCl, pH 8, 10 mmol/L NaF, 1 mmol/L Na3VO4) supplemented with protease-inhibitor-cocktail (Roche/Merck, Darmstadt, Germany). After 2–3 freeze and thaw cycles the protein content of the lysates was measured by using a Bio-Rad DC™ Protein Assay. 50–100 µg of total proteins were used for SDS-PAGE electrophoresis as previously described [19, 76]. The CAV1 (D46G3; #3267) and p21 Waf1/CIP1 (12D1; #2947) antibodies were from Cell Signaling Technology (Danvers, MA; all 1:1000), the Lamin B1 (#12987-1-AP) antibody was from Proteintech Group (Rosemont, IL) and the beta-actin antibody (AC-74, A2228; 1/10.000) was from Sigma-Aldrich. Representative blots from at least three independent experiments are shown.

### Histology and immunofluorescence

Immunohistochemistry (IHC) and immunofluorescence staining was performed on formalin-fixed and paraffin-embedded spheroids as previously described [57]. Fixed (with 4% para-formaldehyde/phosphate buffered saline (PBS) for 30 min) and agarose embedded spheroids were subjected for paraffin embedding and sectioning (3–5 µm). Sections were stained with a PAS staining kit (all Carl Roth Karlsruhe, Germany) according to the manufacture’s protocols for histological evaluation. Prior immunofluorescent staining, samples were prepared by using a descending alcohol series and incubation with target retrieval solution (DAKO/Agilent Technologies Santa Clara, CA). After blocking with 5% normal goat serum (in PBS) for 30 min, primary antibody staining (1/100 dilution) in blocking buffer was performed for 1 h at room temperature (cells) or overnight at 4 °C (paraffin-sections). Nuclei were counterstained using 1 µg/ml Hoechst 33342 (in PBS) for 10–15 min after secondary antibody staining using goat anti-rabbit or goat anti-mouse IgG H&L (Alexa Fluor® 647; dilution 1:500 in blocking buffer). Samples were mounted with Fluoromount-G™ Mounting Medium (Thermo Fisher Scientific). The phospho-NFkB p65 (Ser529) antibody was from Invitrogen (Thermo Fisher Scientific; #44-711G). Nuclei were counterstained with Hoechst 33342.

### Single cell RNA sequencing analysis

Single cell suspensions were generated by re-suspending spheroids in TrypLE (Thermo Fisher Scientific; #12604013) containing 100 u/ml DNase I followed by a 15-min incubation at 37 °C. Digestion was stopped by adding PBS containing 5% FCS, 2 mM EDTA and DNAse I. The cellular solution was passed through a 70 µm cell strainer into a fresh conical tube prior single cell analysis. For scRNAseq, dissociated lung spheroids (single cells) were labeled using the BD® Hu Single-Cell Multiplexing Kit (BD, Bioscience; #633781) according to the manufacturer’s instructions and processed as previously described [72]. In brief, cells (each sample) were stained with Calcein AM to count live cells and DRAQ7™ to count dead cells in a disposable hemocytometer using the BD Rhapsody™ Scanner. Only cell suspensions with viabilities > 80% were used. Desired cell numbers of different samples were combined (Sample 1 CTRL + Sample 1 XRT and Sample 2 CTRL + Sample 2 XRT) and loaded on one lane on a BD Rhapsody HT Xpress System aiming for 40.000 cells. Cell duplet rates were usually ≤20%. BD Rhapsody library preparation was performed according to the manufacturer’s instructions and sequenced on a NextSeq 2000 P3 100 cycles. BDs cwl-runner was used to align and pre-analyse the data. The produced rds file were then used for further analysis. All further analyses were conducted with R packages (Seurat v.4, Enhanced Volcano, scPubr, fgsea, nVennR). Cells with no or more than one sample sag and cells with less than 1500 genes expressed (=0.01%) or more than 10% mitochondrial RNA molecules were excluded. Sample tag filter ensures that multiplets which had more than one cell in a well were removed. The number of genes and mitochondrial filter removes cells, which may have been leaky or dead. Initial clustering was performed with the standard settings and after identification, cell type-specific genes were checked if this is consistent with different resolutions. Standard settings of Cluster identification in Seurat are 0.8.

### Statistical analysis

If not otherwise indicated (*n* = biological replicates), data were obtained from at least from 3 independent experiments. Data were presented as mean values ± SD or SEM (Figs. 1–3) or interquartile range (IQR; Fig. 7) as indicated. Data analyses were performed using Prism 8.0 software (GraphPad, La Jolla, CA) or by two-sided Mann–Whitney U tests in case of unpaired data or by Wilcoxon signed-rank test using R (R Core Team). Statistical significance was set at the level of *p* ≤ 0.05.

## Supplementary information

Supllemental Figures

Supplemental Table 1

Supplemental Table 2

Supplemental Table 3

Supplemental Movie 1

Supplemental Movie 2

Supplemental Figure uncropped gels

## Data Availability

The RNA-seq data (single cell) have been deposited at Gene Expression Omnibus (GEO) and are publicly available as of the date of publication (accession number: GSE297341). Any additional information required to reanalyze the data reported in this paper is available from the corresponding author upon request.
