# Supplementary material for: Senescent fibroblasts modulate the radiation response of neighboring epithelial cells
Source: Cell Death Discov. 2025 Oct 20;11:468. doi: 10.1038/s41420-025-02796-z (PMC12537977; doi:10.1038/s41420-025-02796-z)
Supplement: Supplementary file 1 — Supllemental Figures [file 41420_2025_2796_MOESM1_ESM.pdf]

Supplemental Material

Supplemental Movies

Supplemental Movie 1

Continuous live cell imaging of plated HBEC cells (2000 cells/well) along with green-labeled HS5 fibroblasts (250 cells/well; cyan fluorescent protein, CFP) was performed 1–2 hours after cell plating, after the cells had settled. Spheroids formation was recorded within another 48 hours. The videos depict the continuous phase contrast recording together with the CFP signal (overlay) derived from the fibroblasts alone (CFP-FIB).

Supplemental Movie 2

Continuous live cell imaging containing plated HBEC cells (2000 cells/well) together with HS5 fibroblasts (250 cells/well) labeled in green (cyan-fluorescent protein, CFP) was performed starting 48 hours after cell plating and thus spheroid generation for additional 72 hours. The videos depict the continuous phase contrast recording together with the CFP signal (overlay) derived from the fibroblasts alone (CFP-FIB).

Supplemental Tables (provided as exel file)

Supplemental Table S1

Differentially expressed gene analysis between each cluster and all other clusters to visualize genes that are important for each cluster. All identified genes are shown, even if they were not significantly different between clusters.

Supplemental Table S2

Differentially expressed gene analysis between control and irradiated samples. All identified genes are shown, even if they were not significantly different between the treatments.

Supplemental Table S3

RT-induced genes (down regulated in Ctrl) in lung spheroid meta clusters. Genes differentially expressed following RT in alveolar, airway, and FIB meta clusters.

Supplemental Figures

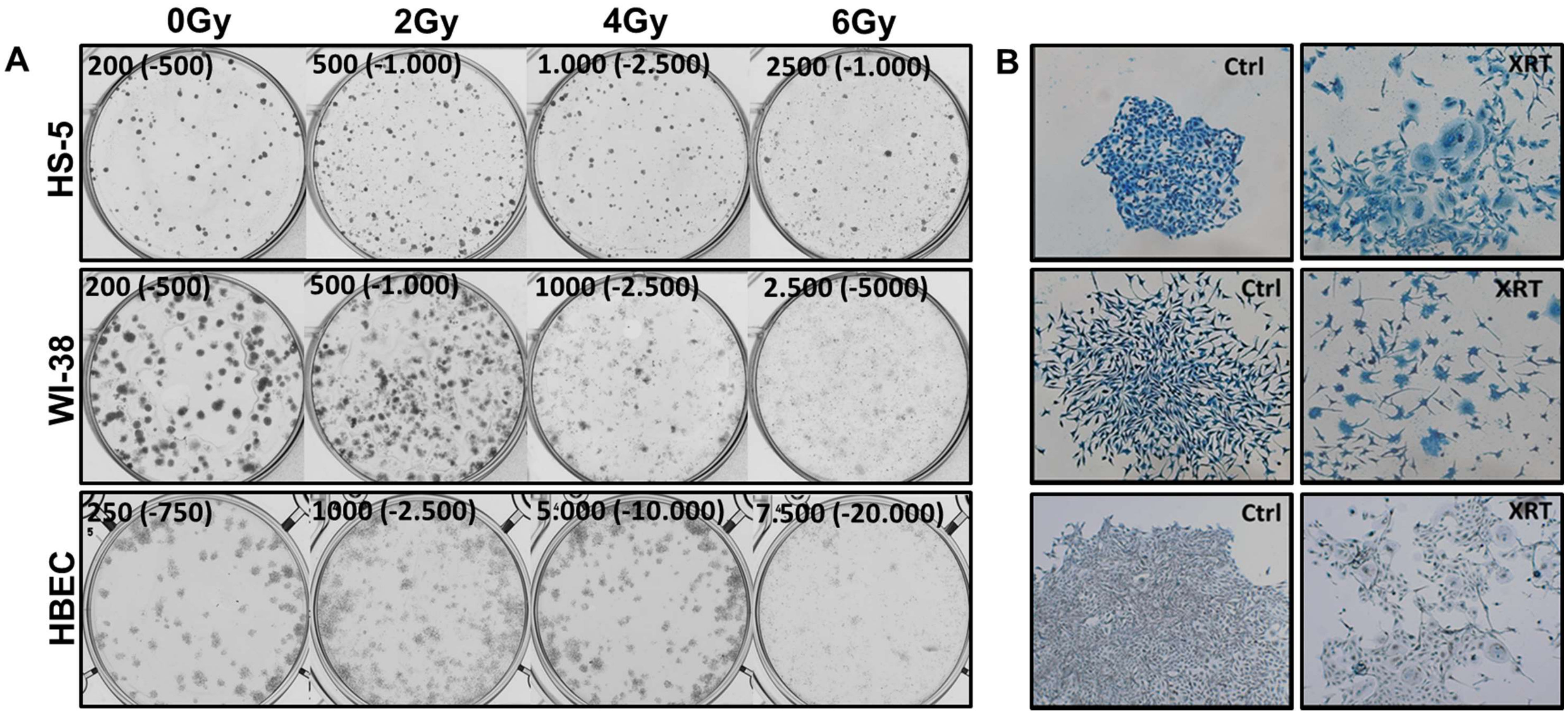

Supplemental Figure S1

Clonogenic survival assay. Cells were plated at low densities (100-1000 cells per well; triplicates) irradiated with indicated doses (2, 4, 6 Gy), and after a 10 days' culture, the cells were washed with PBS, fixed with 4% (w/v) paraformaldehyde, and subsequently stained with 0.05% Coomassie Brilliant Blue. Colonies (⩾50 cells/colony) were counted, and plating efficiencies as well as survival rates were calculated (shown in Figure 1F). (A) The 6-well plates were scanned; exemplary wells of the indicated cell types are shown. Numbers indicate plated cell numbers and the range. (B) Representative colonies for 0Gy (Ctrl) and 6Gy (XRT) are exemplarily shown. Magnification: 10x.

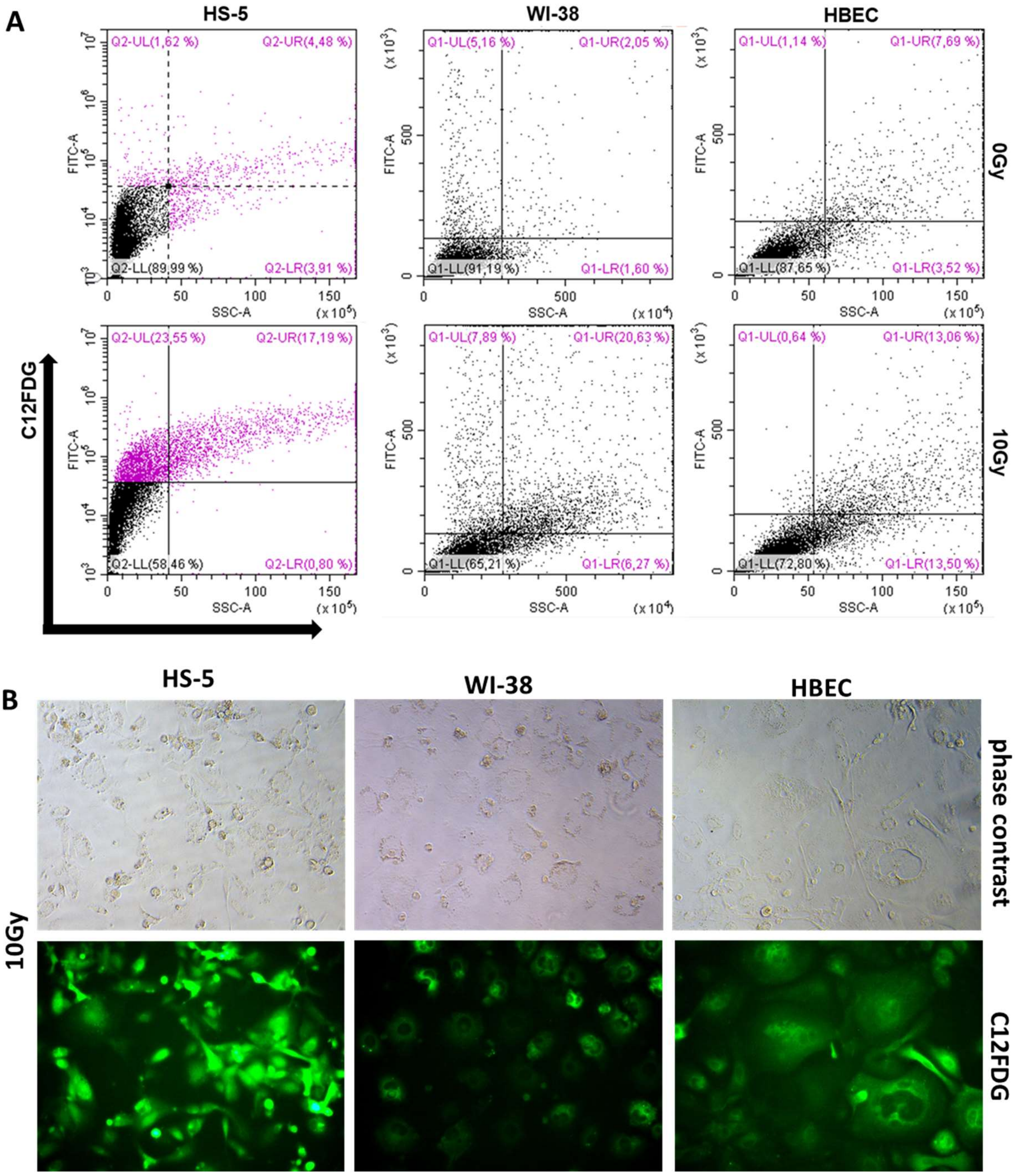

Supplemental Figure S2

RT-induced senescence in 2D cultured fibroblasts and epithelial cells. (**A**) RT-induced senescence formation was analyzed by C12FDG staining of 2D cultured fibroblasts (HS-5 and WI-38) and epithelial cells (HBEC) prior flow cytometric analyses at 96 hours post treatment with 10 Gy. Representative plots obtained from the flow cytometry measurements are exemplarily shown. Senescence levels were determined by the increase in the C12FDG (FITC) signal and/or morphological alterations in the upper left (UL), upper right (UR) and lower right (LR) quadrant as exemplarily highlighted in purple color in the HS-5 plots. (**B**) Senescent and thus C12FDG stained cells (depicted in green) were additionally recorded by fluorescent (and phase contrast) microscopy. Representative pictures are shown.

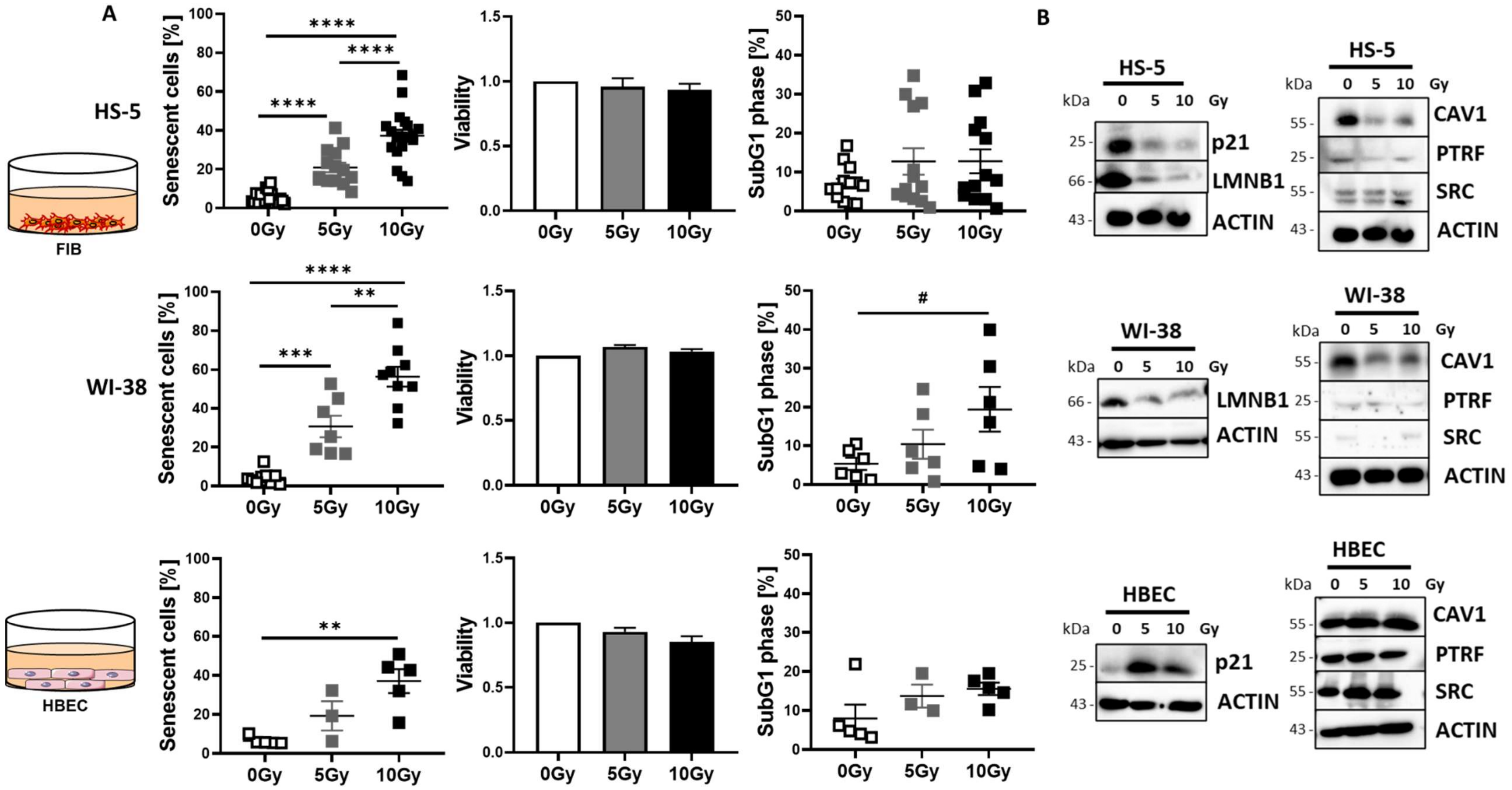

Supplemental Figure S3

Dose-dependency of RT-induced senescence in 2D cultured fibroblasts and epithelial cells. (**A**) RT-induced senescence formation (first panels) was analyzed in 2D cultured fibroblasts (HS-5 and WI-38) and epithelial cells (HBEC) at 96 hours post treatment with 0, 5 and 10 Gy. Individual symbols represent different biological replicates from independent experiments. P by two-way ANOVA, followed by post hoc Sidak's multiple comparisons test: **P ≤ 0.01, ***P ≤ 0.005, ****P ≤ 0.001. Viabilities (middle panels) were analyzed using the WST-1 reagent. Estimated values were related to respective control treatment (0Gy; set as 1). Data represent mean values ± SEM from 4-5 independent experiments measured in quadruplets each. Apoptotic (subG1) cell fractions (right panels) were analyzed by flow cytometry. P by unpaired (two-tailed) t-test depicted as # p ≤ 0.05. (**B**) Expression levels of the indicated proteins were analyzed in whole protein lysates following RT (96 hours) using Western blot analysis. Beta-actin was included as loading control. Representative blots from 3-4 independent experiments are shown.

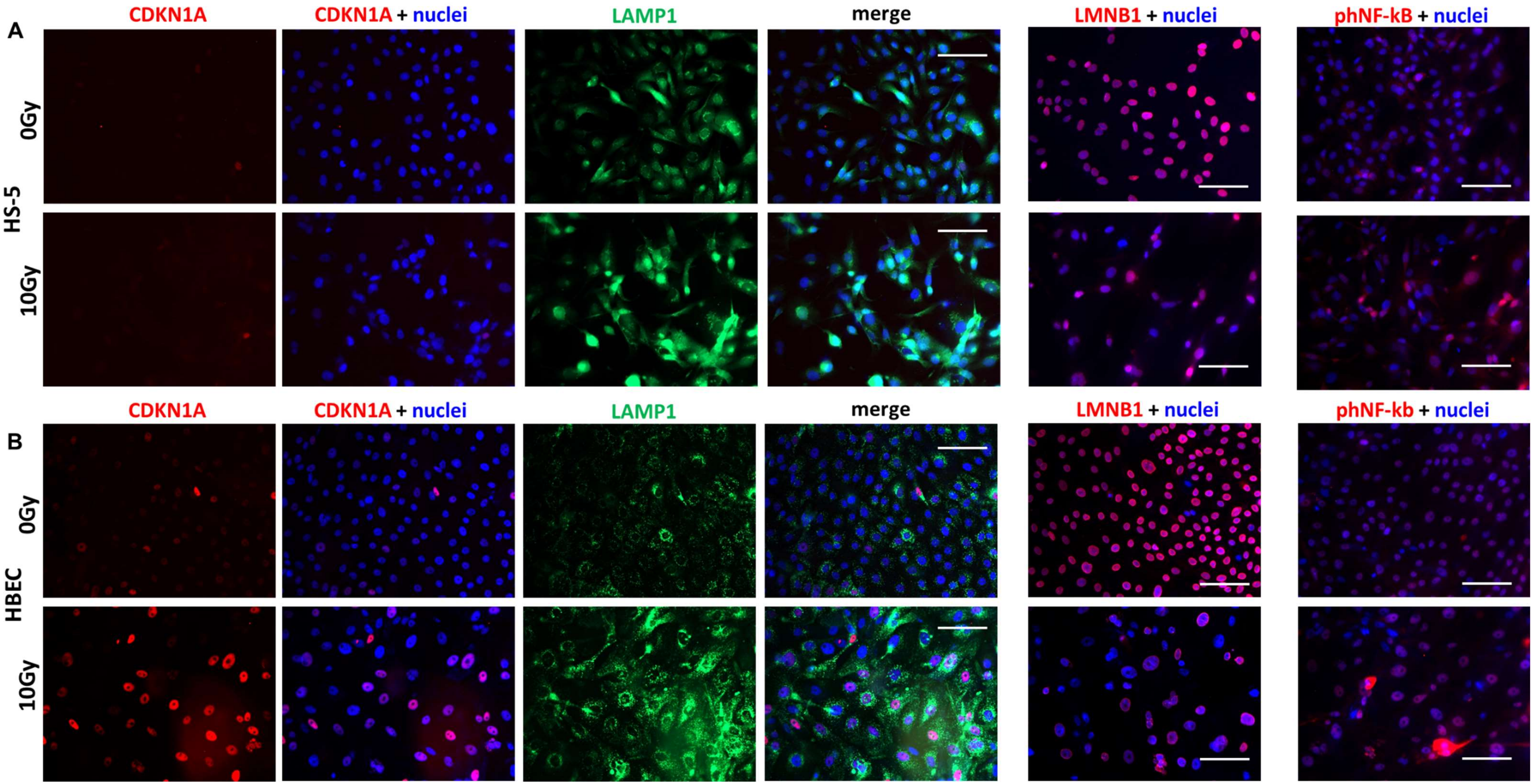

Supplemental Figure S4

Immunocytochemical analysis of the senescence markers. CDKN1A (depicted in red), LAMP1 (depicted in green) as well as LMNB1 and phosphorylated of NF-κB p65 (both depicted in red) were stained via immunofluorescence in 2D cultured HS-5 fibroblasts (**A**) and HBEC epithelial cells (**B**) at 96 hours post irradiation treatment with 10 Gy. Representative images are shown. Nuclei were stained with Hoechst 33342 (blue). Magnification: 63x.

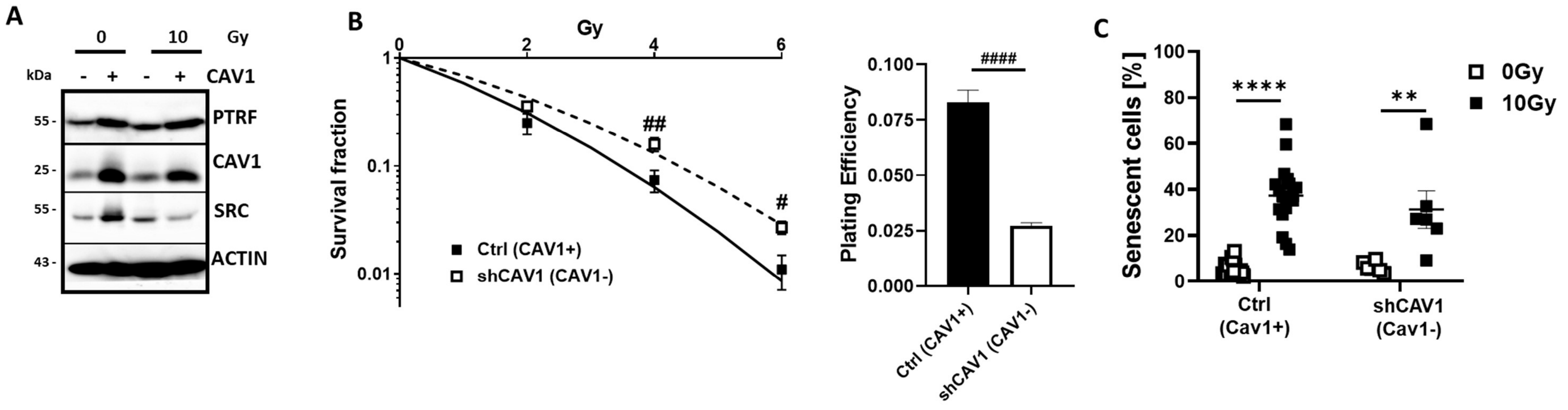

Supplemental Figure S5

Reduction of CAV1 levels increased clonogenic survival of HS-5 fibroblasts while senescence induction was not affected. (**A**) Lentiviral expression of a CAV1-specific siRNA (shCAV1) in stromal HS5 fibroblasts resulted in an efficient and sustained down-regulation of CAV1 expressions compared to control-transduced (Ctrl) cells as shown by Western blot analysis. Expression levels of the indicated (CAV1-associated) proteins were additionally analyzed in whole protein lysates following radiation treatment (96 hours after RT with 0 10Gy). Beta-actin (bActin) was included as loading control. Representative blots are shown. (**B**) HS-5 fibroblasts with differential CAV1 levels were plated for colony formation assay, irradiated with indicated doses (0–6 Gy) and subsequently further incubated for additional 10 days. Data show the surviving fractions (left) and the plating efficiencies (PE, right) from three independent experiments measured in triplicates each (means ± SD). P by unpaired (two-tailed) t-test depicted as # $p \leq 0.05$, ## $p \leq 0.01$, #### $p \leq 0.001$. (**C**) RT-induced senescence formation was analyzed by C12FDG staining prior flow cytometric analyses at 96 hours post treatment with 10 Gy. Graph depict data from 3-5 independent experiments. Individual symbols represent different biological replicates. P by two-way ANOVA, followed by post hoc Sidak’s multiple comparisons test: **$p \leq 0.01$, ****$p \leq 0.001$.

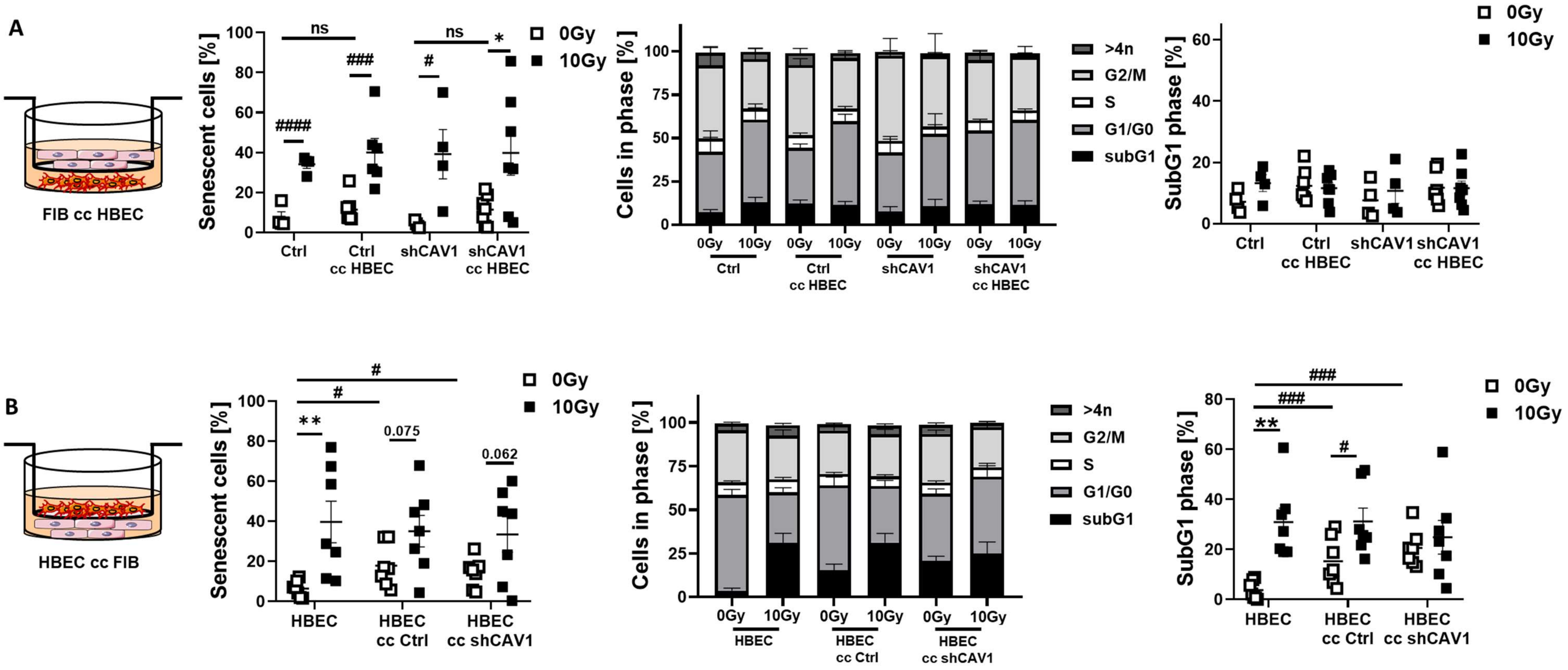

Supplemental Figure S6

Differential CAV1-levels in fibroblasts do not affect RT-induced senescence in HBEC transwell co-cultures. (**A**) CAV1-silenced (shCAV1) or control (Ctrl) HS-5 were cultured alone ('empty' transwell) or together with HBEC in transwells (cc HBEC) for 24 hours prior to RT with 0 or 10 Gy, and analyzed after additional 96 hours. Senescence induction, cell cycle phases and apoptotic cells (subG1) were analyzed by flow cytometry. (**B**) In the other way round, HBEC were cultured alone ('empty' transwell) or together with HS-5 (cc HS-5) differentially expressing CAV1 for 24 hours prior to RT with and analyzed after 96 hours post RT. Senescence, cell cycle phases and apoptotic cells were determined. Individual symbols represent different biological replicates. P by two-way ANOVA, followed by post hoc Sidak's multiple comparisons test: $^{**}p \leq 0.01$ and additionally by unpaired (two-tailed) t tests depicted as # $p \leq 0.05$, ## $p \leq 0.01$, ### $p \leq 0.005$; ns not significant.

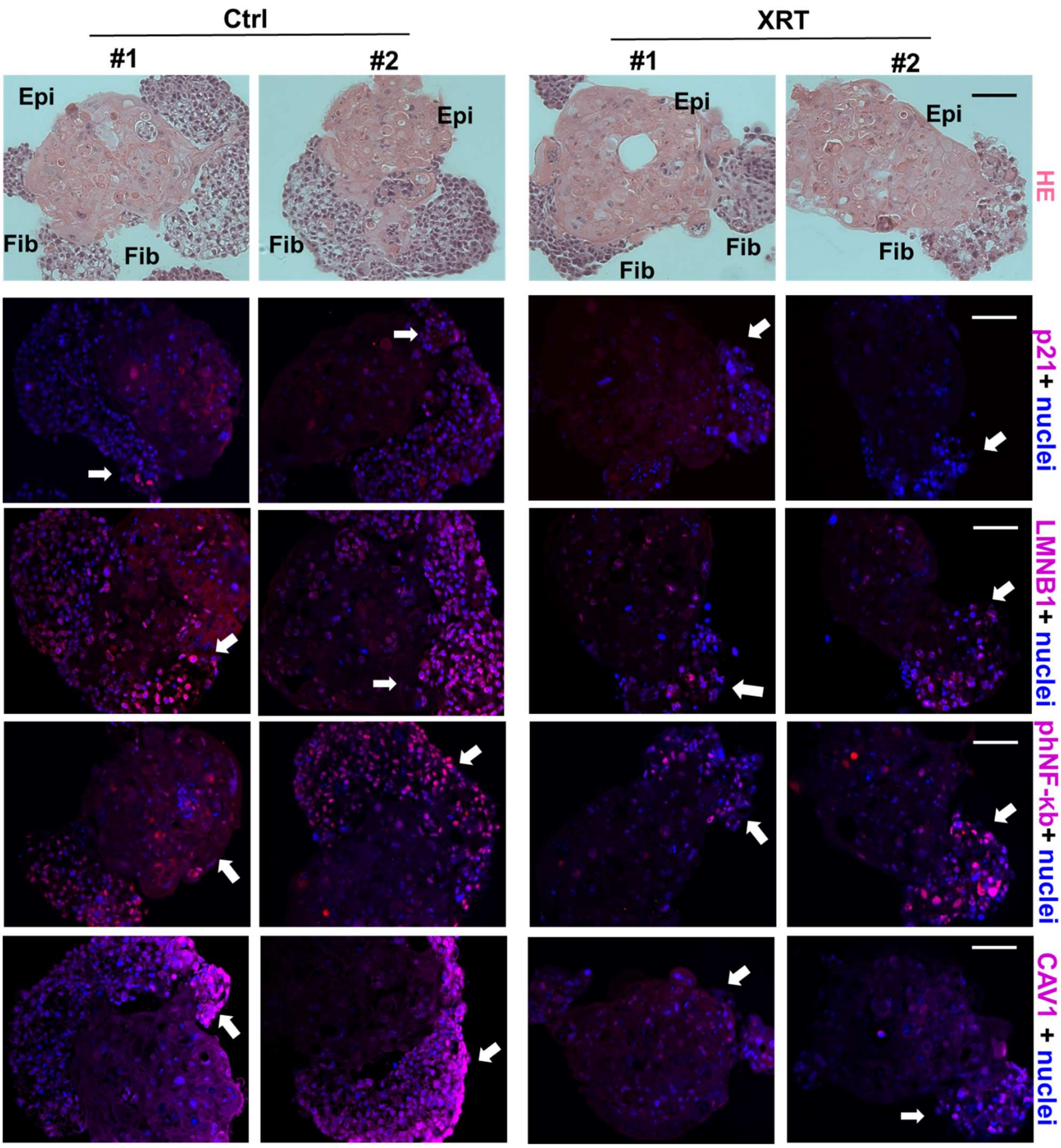

Supplemental Figure S7

Immunohistochemical analysis of the senescence markers. Hematoxylin and Eosin (HE; upper panel) histology staining of paraffin-embedded sections of spheroidal co-cultured HS-5 fibroblasts and HBEC cells at 96 hours following radiation treatment with 10 Gy. Magnification: 20x. Immunofluorescence staining of successive sections were performed using the senescence markers CDKN1A, LMNB1 and phosphorylation of NF-κB p65, as well as the protein CAV1 (all depicted in purple). # Numbers indicate different biological replicates. Arrows point toward immune-reactive structures in fibroblasts (FIB). Epi, epithelial cells. Nuclei were stained with Hoechst 33342 (blue). Representative images are shown. Magnification: 20x.

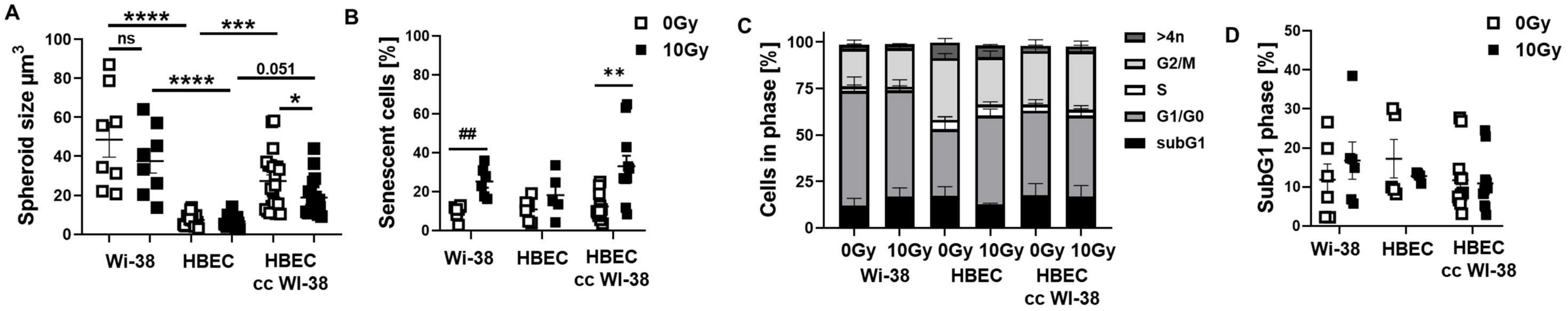

Supplemental Figure S8

Radiation-induced senescence in WI-38 fibroblasts and HBEC epithelial spheroidal co-cultures. (**A**) WI-38 and HBEC cells were cultured as spheroids either alone (2000 cells per spheroid) or together (Wi-38 cc HBEC; 2000 HBEC cells together with 250 Wi-38 fibroblasts per spheroid). Spheroid growth was measured at 96 hours post RT with 10 Gy and respective volumes were calculated. Individual symbols represent different biological replicates. (**B**) Senescence levels, (**C**) distribution of cell cycle phases and (**D**) apoptotic cells were determined in whole spheroids following generation of single cell suspensions. P by two-way ANOVA, followed by post hoc Tukey's multiple comparisons test: *p ≤ 0.05, **p ≤ 0.01 ***p ≤ 0.005 ****p ≤ 0.001 and additionally by unpaired (two-tailed) t tests depicted as # p ≤ 0.05.

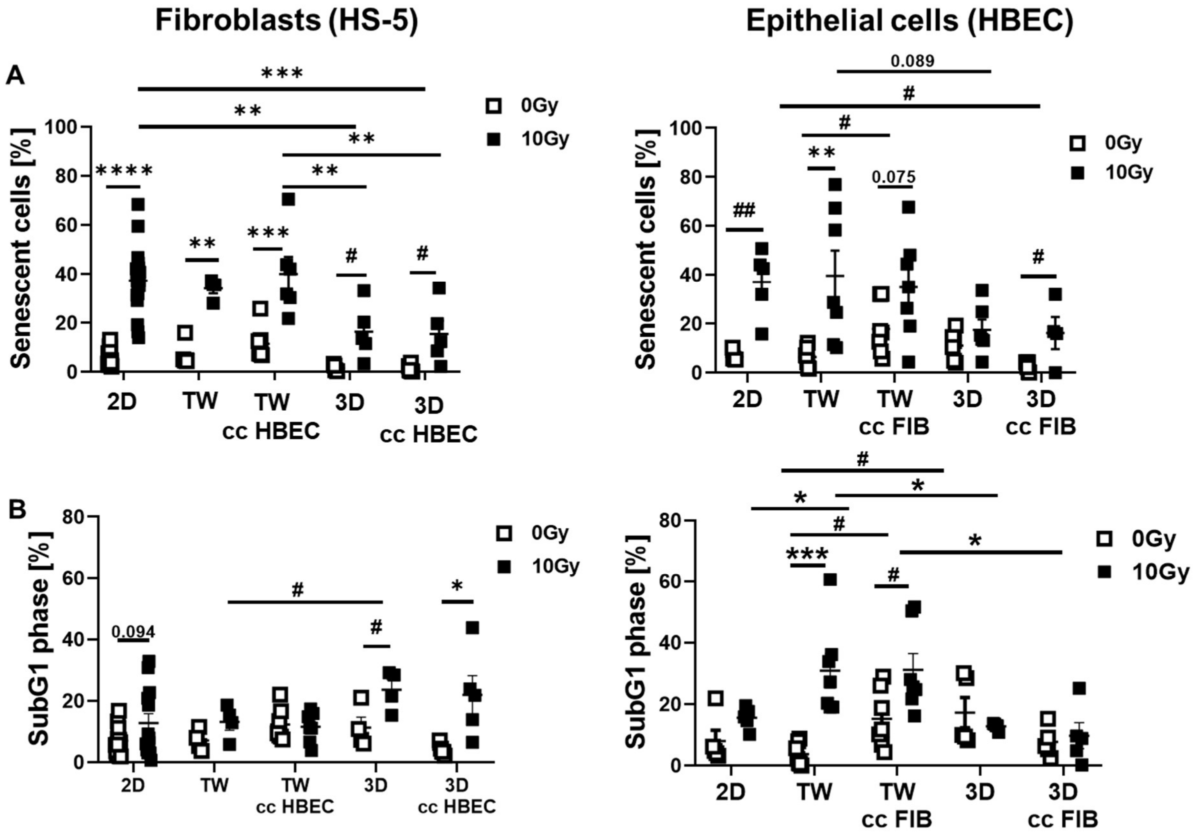

Supplemental Figure S9

Comparison of RT-induced senescence and apoptosis induction using different cultivation methods. HS-5 and HBEC cells were cultured either alone classically as flat monolayers (2D), together with the other cell type in transwells (TW cc) or using empty transwells as controls (TW), or as spheroids either alone (3D) or in co-culture (3D cc). RT-induced senescence (**A**) and apoptosis induction (subG1 fractions) (**B**) were determined for HS-5 fibroblasts and HBEC epithelial cells at 96 hours pot RT with 10Gy. Individual symbols represent different biological replicates. P by two-way ANOVA, followed by post hoc Sidak's multiple comparisons test: *p ≤ 0.05, **p ≤ 0.01 ***p ≤ 0.005 ****p ≤ 0.001 and additionally by unpaired (two-tailed) t tests depicted as # p ≤ 0.05, ## p ≤ 0.01.

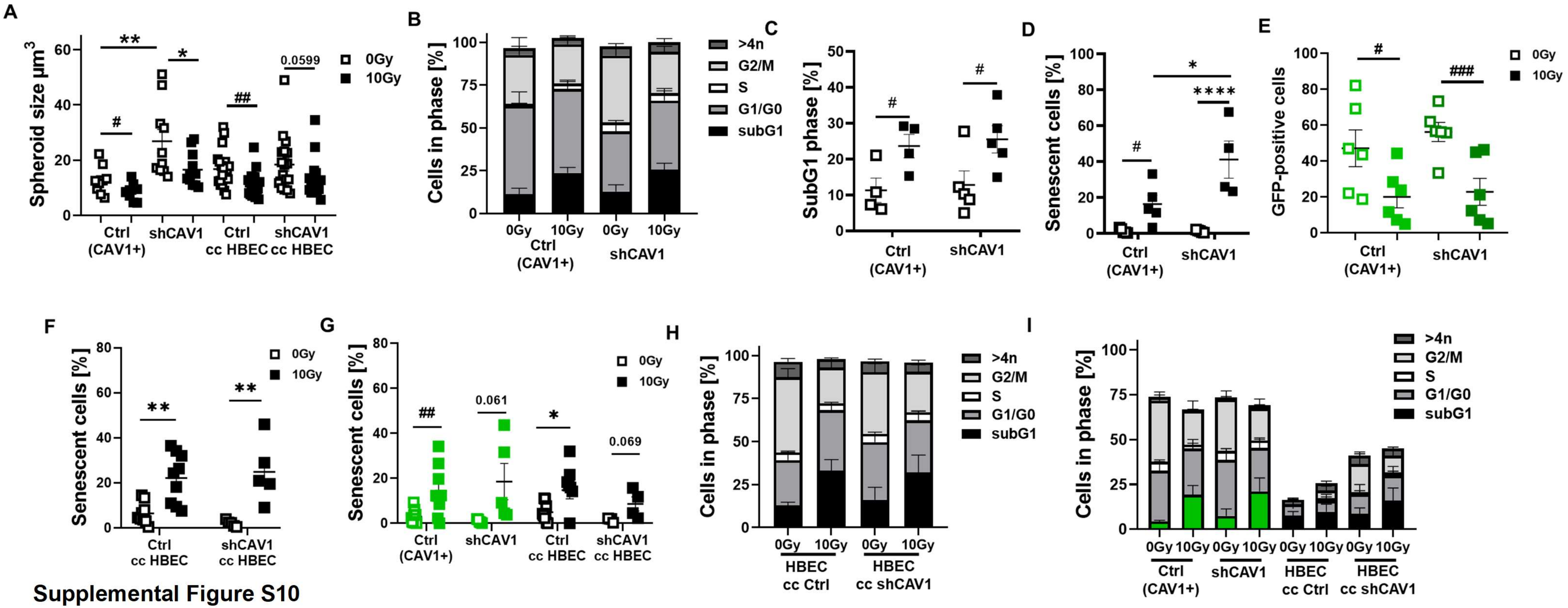

Supplemental Figure S10

CAV1-silenced fibroblasts do not impact on RT-induced cell fates in spheroidal co-cultured HBEC cells, although RT-induced senescence levels were increased in pure CAV1-silenced fibroblast spheroids. HS-5 fibroblasts, either CAV1 proficient (CAV1+) or CAV1 silenced (shCAV1), and HBEC cells were cultured as spheroids either alone (2000 cells per spheroid) or together (HS-5 cc HBEC; 2000 HBEC cells together with 250 HS-5 fibroblasts per spheroid). (**A**) Spheroid growth was measured at 96 hours post RT with 10 Gy and volumes were calculated. (**B**) Distribution of cell cycle phases, (**C**) apoptotic and (**D**) senescent cells were determined in whole spheroids generated from single cell suspensions. (**D**) The numbers of CFP-expressing cells (fibroblasts) were determined by flow cytometry (96 hours post RT) based on the labelling. (**F, G**) RT-induced senescence and (**H, I**) distribution of cell cycle phases including apoptotic cells were determined in whole spheroids. (**G, I**) Respective signals were additionally related to the CFP-expressing fibroblasts (indicated by green color). Individual symbols represent different biological replicates. (**A, C-G, I**). P by two-way ANOVA followed by post hoc Tukey's multiple comparisons test: *p ≤ 0.05, **p ≤ 0.01, ****p ≤ 0.001 and additionally by unpaired (two-tailed) t tests depicted as # p ≤ 0.05, ## p ≤ 0.01 and ### p ≤ 0.005.

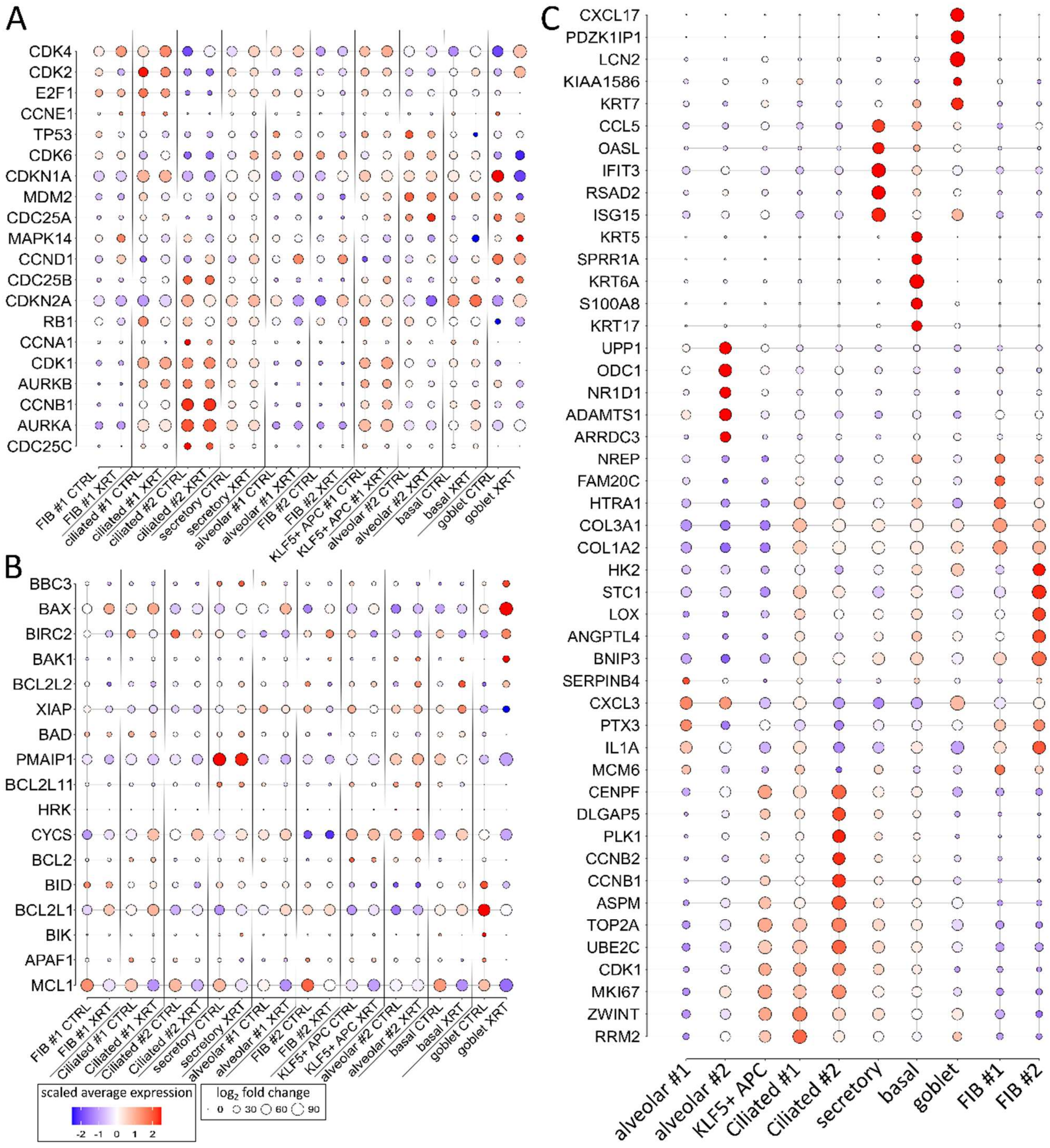

Supplemental Figure S11

Gene expression alterations of lung spheroids in response to RT as obtained from scRNAseq data sets. (**A**) Expression of cell cycle genes in each cluster before and after irradiation (XRT). (**B**) Expression of apoptosis-related genes in each cluster. (**C**) Dot plot of the top five differentially expressed genes per cluster after irradiation. Size of the dots indicate the percentage of cells in which this gene was found, the color indicates the normalized value of the expression.
