# Supplementary figures and images for: Senescent fibroblasts modulate the radiation response of neighboring epithelial cells
Source: Cell Death Discov. 2025 Oct 20;11:468. doi: 10.1038/s41420-025-02796-z (PMC12537977; doi:10.1038/s41420-025-02796-z)

Figure S1B HS-5

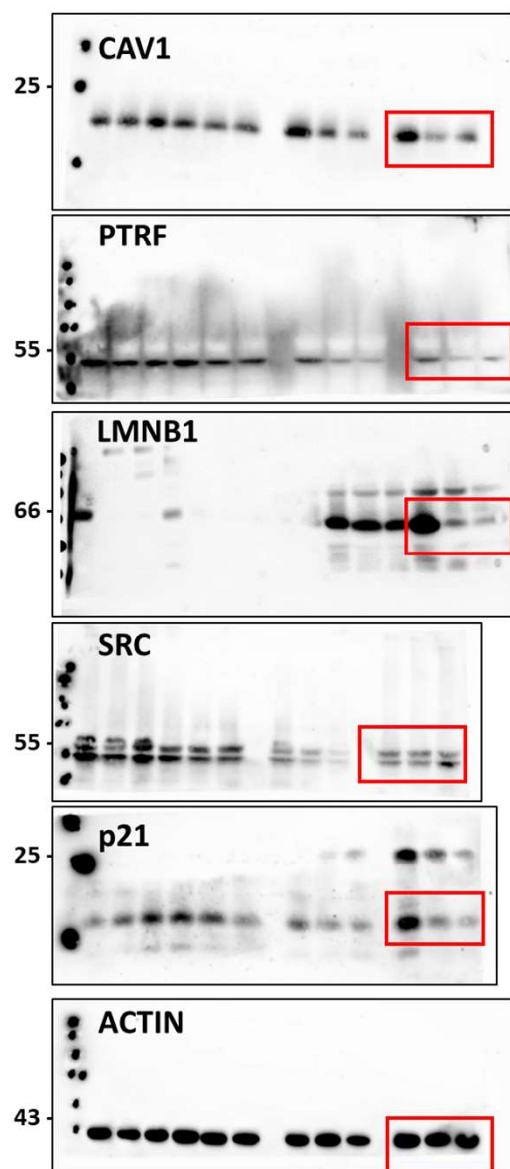

Figure S1B WI-38

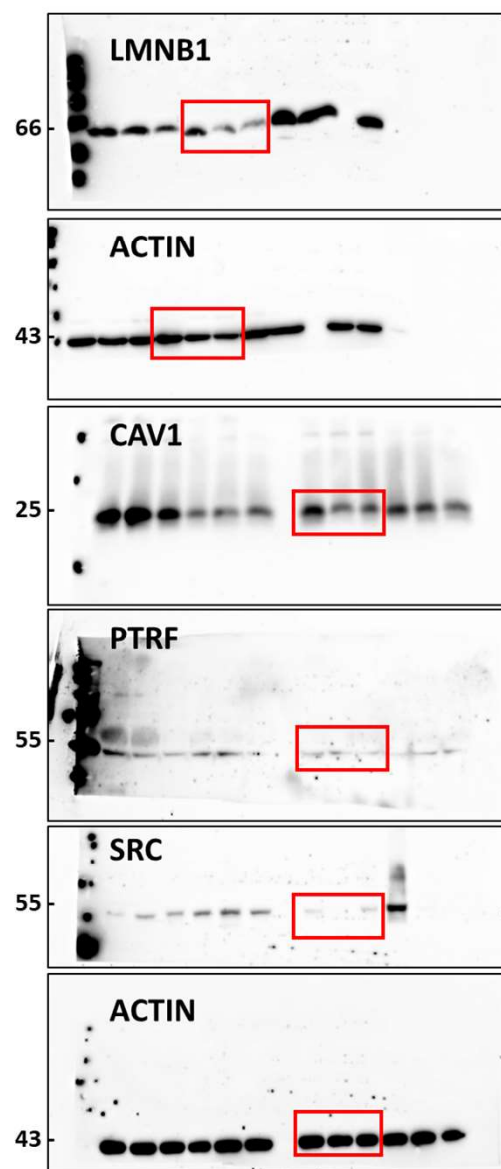

Figure S1B HBEC

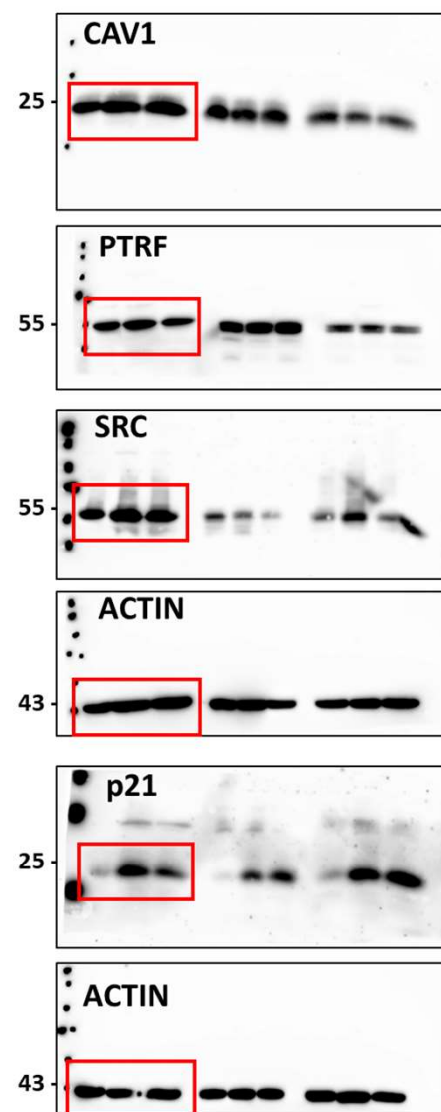

Figure 2

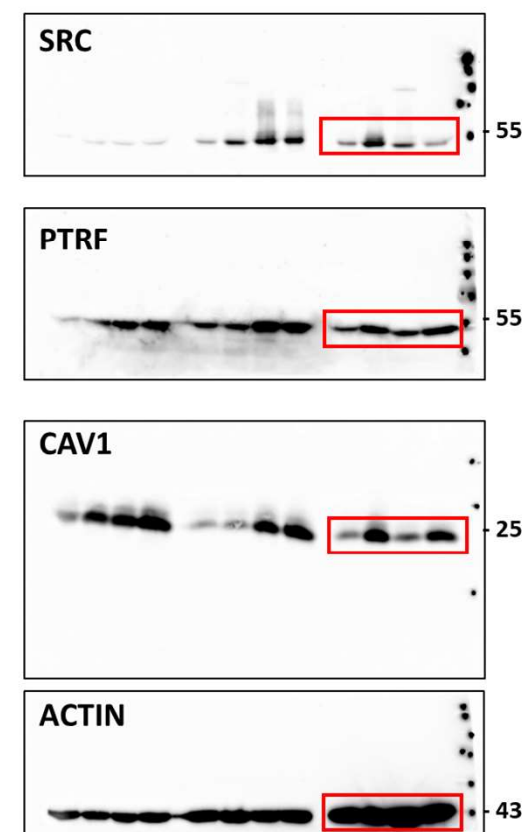

Supplement: Supplementary file 7 — Supplemental Figure uncropped gels [file 41420_2025_2796_MOESM7_ESM.pdf]
